# Supplementary material for: Health professionals’ experience on District Health Information System (DHIS2) and its utilization at local levels in Gandaki province, Nepal: A qualitative study
Source: PLOS Glob Public Health. 2024 Mar 27;4(3):e0002890. doi: 10.1371/journal.pgph.0002890 (PMC10971587; doi:10.1371/journal.pgph.0002890)
Supplement: S1 Text — (DOCX) [file pgph.0002890.s002.docx]

**IDI guide for local level focal person**

**In-depth Interview Guideline for Local Level DHIS2 Focal Person**

Name of local level:

Current position:

Year of experience on DHIS2:

1. What are your experiences in using DHIS2 at your Palika?
2. What is the current status of DHIS2 at your Palika?
3. How has DHIS2 impacted your work?

**(Probe:** Recording, reporting, analysis, Monthly/quarterly review meeting, Data quality checks, health service delivery, Performance evaluation using DHIS2)

1. How is the data from DHIS2 utilized at your Palika?

**(Probe on:** Service coverage, disease trends, Monitoring and supervision and quarterly review meeting, Development of local plans, Annual work plan and budgeting)

1. What motivates you to use DHIS2 at your Palika?
2. What are the challenges experienced while using DHIS2?

**(Probe on:** By you and other staffs at health facility)

1. How have you addressed those challenges?
2. What are the opportunities in using DHIS2?
3. Who provides you the feedback regarding the use of DHIS2?

(**Probe:** Province level, District level)

1. What training/workshop have you received/attended on DHIS2?

(**Probe:** Basic training, Refresher training, Training materials, training needs)

1. How interested are the elected representatives, administrative personnel, and other stakeholders in DHIS2?
2. What comments or suggestions that you would like to add to help us understand DHIS2 utilization in Gandaki Province?
