# Supplementary material for: Health professionals’ experience on District Health Information System (DHIS2) and its utilization at local levels in Gandaki province, Nepal: A qualitative study
Source: PLOS Glob Public Health. 2024 Mar 27;4(3):e0002890. doi: 10.1371/journal.pgph.0002890 (PMC10971587; doi:10.1371/journal.pgph.0002890)
Supplement: S2 Text — (DOCX) [file pgph.0002890.s003.docx]

**IDI guide for health post focal person**

**In-depth Interview with Health facility Focal Person**

Name of local level:

Current position:

Years of experience using DHIS2:

1. How have you been involved in using DHIS2?

(Probe: What type of data do you need to enter?)

1. What is the current status of DHIS2 at your institution?
2. What are your experiences in using DHIS2?

**(Probe:** Efficiency of recording and reporting, Data generation and dissemination, Monthly review meeting, Monitoring and evaluation, Data quality checks (input validation, use appropriate ranges and validation rules)

1. How is the data from DHIS2 utilized at your health facility?

**(Probe:** Service coverage, disease trends over time, Feedback meeting and quarterly meeting, Decision-making process, performance evaluation)

1. How has DHIS2 impacted the quality of healthcare services provided at your workplace?
2. How local levels are utilizing DHIS2 for various actions?
3. What motivates you to use DHIS2?
4. What are the challenges experienced while using DHIS2?

(Probe: During data entry and reporting, analysis, data validation, error management, support from authorities)

1. How have you addressed those challenges?
2. What are the opportunities in using DHIS2?
3. Have you received any training/workshop on DHIS2?

(**Probe:** Basic training, refresher training, training needs, training manuals)

1. Who provides you the feedback regarding the use of DHIS2?

(Probe: Local level, district, local level)

1. What are the potential areas for improvement in DHIS2 and its utilization?
