# Supplementary material for: Health professionals’ experience on District Health Information System (DHIS2) and its utilization at local levels in Gandaki province, Nepal: A qualitative study
Source: PLOS Glob Public Health. 2024 Mar 27;4(3):e0002890. doi: 10.1371/journal.pgph.0002890 (PMC10971587; doi:10.1371/journal.pgph.0002890)
Supplement: S3 Text — (DOCX) [file pgph.0002890.s004.docx]

**IDI guide for province focal person**

**IDI guideline for Provincial Health Directorate DHIS2 Focal Person**

Current position:

Years of experience on using DHIS2:

1. What is the current situation of DHIS2 utilization in Gandaki province?
2. What are the major challenges experienced by DHIS2 users at local level and health facility level?
3. How have you been addressing these challenges?
4. What have you seen the possible opportunities for utilization of DHIS2 at local level?
5. How do you supervise and monitor DHIS2 and its utilization below the province level?
6. In your experiences, what are the possible strategies or suggestions for better DHIS2 utilization at Gandaki province?
