# Supplementary material for: Health professionals’ experience on District Health Information System (DHIS2) and its utilization at local levels in Gandaki province, Nepal: A qualitative study
Source: PLOS Glob Public Health. 2024 Mar 27;4(3):e0002890. doi: 10.1371/journal.pgph.0002890 (PMC10971587; doi:10.1371/journal.pgph.0002890)
Supplement: S4 Text — (DOCX) [file pgph.0002890.s005.docx]

**In-depth Interview Transcripts - Local Level DHIS2 Focal Person**

1. **Interview: Fedikhola rural municipality DHIS-2 focal person**

Fedikhola Rural Municipality

Sr. ANM

Female

Years of experience using DHIS2: 3 years

I: What are your experience on using DHIS-2 in this rural municipality?

R: It has almost been 3 years since we are using DHIS-2. At first we do find this system a new one and we didn’t use it. In earlier time we do use it for data entry only. During data entry, we thought that the color changes denotes that data had entered but after completing the data entry it has been unavailable or data was not shown. So, we faced such problems. But now it is so easy to use this system because whenever and wherever data can be used for presentations. Monthly, quarterly and annual data can be operated easily. But sometimes, we faced problems like “system errors” and “unreachable” and we have to complete the reporting by 15^th^ in every month.

One of my experience is, I have entered data for 3 times. After the completion of entry signal is shown, I was sure of completing the entry but after opening the system again after certain time we finded it empty and unable to see the entered data. I don’t know what had happened at that time but due to which we were late in data entry.

We usually enter data in time. If I talk about my working schedule then, I have entered data from home after office time. Usually, server get busy in office time so I have entered data for 10, 11 PM at night. But when I show my entered data next morning to senior staffs then due to error data had been deleted. Those are our errors.

I: Right now, what types of data are we entering?

R: We enter data according to the new and old patients’ program which ranges from health problems to mental health problems. And yeah! We also enter data of COVID-19 vaccination in DHIS-2.

I: Has DHIS-2 reached in every municipality’s health facilities?

R: After receiving training related to DHIS-2, data entry is carried out in Fedikhola health post and Arukharka health post. Besides that, we ourselves enter data of one community health unit, 2 health post and 1 primary health care centers here from this municipality.

I: Is timely reporting from health facilities is carried out from respective health facilities?

R: They also face the same problem which I used to have. However, they report on time.

I: Along with data entry, what is the condition of data analysis and data review?

R: What we usually do is, all representative from health institutions come and analyzed together through discussion and then only data entry is carried out. Sometimes error are seen that is different things but mostly errors are not seen.

I: Recently do you record and report completely through DHIS-2 or manually also?

R: We do it both in DHIS-2 and manually also.

I: Do you now analyze data by tallying also?

R: We analyze data even by tallying when in need. As all the data are given by DHIS-2 so we use it.

I: You have been using DHIS-2 for the past 3 years. How it has made easy in your works?

R: It has made very easy and it is easy also because sometimes there might be mistake when tallying by our hands. But even if there may be mistake in DHIS-2, they monitor from here, from district, from province and the error is identified. So, that the system is good.

I: Has it made easy to record, report and analyze?

R: It is easy. We can retrieve data for presentation at any time. So, it has been easy.

I: And in monthly, quarterly or annual meetings or review conduction?

R: It has been almost 2-3 months since we have been discussing by opening DHIS-2 of every health institutions. By opening data, we see what types of data is there and what errors are there. Sometimes we compare DHIS-2 data with the reporting done in paper to identify the mistakes.

I: Do you give suggestion based on this meetings?

R: Oh yes! By seeing the work of one by the other, their mistakes are identified from that.

I: As you said, reporting is carried out in time and the data is available on time. Due to this, has it made easy in providing health care?

R: After analyzing the situation from DHIS-2, it has been easy to provide health care facilities.

I: Has availability of data in DHIS-2 helped in decision making?

R: Yes, it helps.

I: For examples?

R: To do annual review we see data from there. And we make bar diagram or print it and make presentations.

I: For example in any programs?

R: Planning is done according to the data. Data from last FY will be analyzed and planning will be done accordingly for this FY.

For example, safe motherhood is very low and it will be analyzed through DHIS-2 for increment.

I: Do health facilities performance evaluation will be done through DHIS-2?

R: Yes we do. When I am entering data, we open it to see whether other have entered data correctly or not. Likewise, sir overview the data of different institution and checks if someone had not entered data or something is missing in data.

I: How do maintain the quality of the data? Have you seen improvement in data quality?

R: Yes, improvement are seen because different validation are available. For example, we cannot work on it, if it gets locked. If any error happens, they tell immediately. If deworming tablets for pregnant and iron tablets does not matched then DHIS-2 will shows the reason behind difference in the data. Due to which that makes us aware.

I: Do service coverage and disease trend data are analyzed and shared?

R: To share, we have review meetings. Data of 3 years is viewed by opening data in the review meetings. Data of every years is analyzed by using DHIS-2.

I: Data are shown in municipality or shared in any media by elected member of Administration in any organization?

R: We are planning to print flex but it is not done yet. We are having related slides and records of the data. As per the necessity, data can be shown in the elected representative of the Administration and can be shared through email as well.

I: Do you make the use of data while preparing Annual work plan and budget?

R: Yes, looking at the data, How to increase the coverage? How it can be improved? Or Do any additional Programme can make any difference?

I: How you were encouraged to use DHIS-2?

R: Well, it is not troublesome and not necessary to do tallying. If needed, any kind of report at any time can be made. Also if we do have login id and password we can use it whenever needed. It is easy to use and if any sorts of error made it can be known easily. But there is also an issue in the system; there is an option called data visualizer, if I make data entry today sometimes data visualizer is not shown for 12 hours or 24 hours. When I attend the office time, I’d have urge to see the data’s been shown timely or not but sometimes Red signal is shown which means reporting is not done.

So, one thing after entering the data why it is not mentioned or shown in the cell? Sometimes “unreachable” is the issue. Rest of it is fine.

I: What are the general problems reported from the Health Institutions?

R: Same kind of problem such that of “unreachable”,” empty cells” after data entry is seen in Health institutions as well. Sometimes, we will have the enter data at home also. Previously, after entering the data for the 3^rd^ time the data was not shown in the cells. So, we check out time and often if it is entered or not. We even think of not using the tool because of this problem but now it is running well.

I: What are the mistakes while entering the data?

R: Yes, sometimes mistake happens. Often double click leads to fault entry. But, it does not take long enough to enter the data, we can finish entering 1 data within the 15 minutes.

I: What are the challenges while utilizing the data in local settings?

R: No, we don’t find any challenges. It is easier to make use of the data.

I: What is the condition of Internet?

R: If you would visit here previously, you could see the problem of electricity. But now there is delay in reporting of the data while raining. Otherwise, the condition is fine.

I: Do you get help from Provincial and federal Government while using DHIS-2?

R: Yes, they visit time to time and ask about the problem if any. Recently, they came from Province level and once made login id and password for one of our Health Post but later on it was not

Possible make the data entry. Any way they visit time to time.

I: How did you manage that problem?

R: The data is entered again if it is not shown in the cells and the process is repeated until it is done. But in the last month of Shrawn the data couldn’t be entered and shown in time and later the id was locked. But sometimes data brought by Health Institutions in the paper and data entered is different which is later consulted, corrected and finished.

I: Has any effort made from Provincial Government to solve this Problem?

R: We make calls, when we get any kind of Problems. And then they provide instructions accordingly. If we follow the process sometimes the issue is solved next day doing same if not today.

I: What can be the opportunities to make the DHIS-2 tools more effective?

R: a. By providing Refresher training.

b. By solving the internet issues.

c. By visiting the local level frequently by the Provincial Government, also providing support, Guidance, instructions when required.

I: So, what suggestions do you give to improve the DHIS-2?

R: If the internet connection gets better the work can be done smoothly. Also Training to the sufficient workers in the organization from Provincial Government should be provided so that one can do in place of other if needed. There is still the need to make use of DHIS-2 in ¾ organization in our rural municipality.

I: How many times did you attended the Training?

R: I have attended training of 3 days for once. I carry out the work as per it for now.

I: Did you get any kind of Training/Reading material or manual while attending the Training?

R: No, we were not provided any kind of reading material or manual. We took our Laptop and learn from that. It would be better If they provided us with Manual guide while getting any related problems or error we could use it.

The refresher training is not so effective. We couldn’t learn more on that 3day training. It would be better if additional 3 day is added. I feel like it’d be more fruitful.

Also, refresher training should be provided to those who have already attended 3 days training session, so that they can know about the updated and additional things. Eg: COVID-19 data was added later.

I: How is the interest of community representative, administration and other stakeholders on DHIS-2?

R: They are informed about the process of data entry also we take report from this Tool. (And then he started discussion with other members……)

I: Is there any additional suggestions on DHIS-2 would you like to give?

R: This system is good; any missing system can be included to make it better. Some data which are not required eg: Leprosy … should be opened and completed but, we don’t have any problems for now. LMIS system is in place so, it is not so necessary to integrate in DHIS. Overall, the system is good, all data relating to COVID-19 vaccination is also incorporated.

Some Programs relating to Elderly Health Program, Program conducted by local level is not reporting.

Target vs achievement should also be made easier to access. Migrated population from one Palika to other for institutional delivery should be made known so, that target vs achievement is shown and Recording and Reporting can be effective and fine.

1. **Interview of DHIS 2 Focal person of Putalibajar Municipality**

Putalibajar Municipality

Sr. AHW

Male

Years of experience using DHIS2: 4 years

I: Could you please tell me about your experiences while operating DHIS2?

R: I have been using DHIS2 for 4 years. Initially, there was another sir who used to operate it but after he got transferred , then I started operating it. At first , I used to operate without any training for 5-6 months and after that I got training and I am operating it till date.

I: How easy / uneasy is it for you to operate DHIS2?

R: It is not uneasy to operate it, it is quite easy but when we are in a hurry for data entry, at that time server goes down and at that time , it is a little difficult. Server is very busy during reporting times which may have happened as there is a single server nationwide which has made it difficult. Also, in our reporting days all the health institution personnel come here. On that day, while trying to check data of all the health institution, DHIS2 does not function. There is problem in data verification as well.

I: Are there DHIS2 in every health institution of this municipality?

R: Yes, all health institutions have DHIS2 but all do not operate DHIS2 because our 5 health posts do not have internet facility. There is access to computer, they do have user id but they do not have internet. Because of unavailability of internet, they are not able to operate it.

I : How do those health posts currently enter data in DHIS2?

R: They leave their reports in health section of municipality and we do their data entry.

I: How is the situation regarding data analysis and data review ?

R: We are doing that. Staffs from health institutions come in monthly meeting and we do that together.

I: How has DHIS2 made your work easier?

R: It used to be difficult during reporting. Monthly , Quarterly , Annual data generation , data review generation used to be tough but now DHIS2 has made it easier, necessary data are generated in one click. Any data can be generated in a while so it has become easier. Sometimes when it doesn't work, it is difficult then. Any error in the data can easily be corrected.

I: As you said DHIS2 has data generation and review easier , has this made it easier to provide health service?

R: It has helped in planning . For example, elderly program was started at our local level in which data of elderly population present in DHIS2 was used to estimate medicines needed and other things needed were estimated based upon the data of DHIS2 .

I: Have you performed work performance evaluation of health institutions using DHIS2?

R: The reporting of health institution is looked upon and what should have been and how it is going on is seen. In this month why certain things are not achieved and where it is lost is sought for.

I: Is there improvement in data quality because of DHIS2?
R: Compared to previous days, quality has changed. In paper-based reporting, errors for example 56 were mistakenly written as 65 but now it is not the case.

I: Different plans are developed and different decisions are made like annual work plan and budget. On those activities how often is the DHIS2 data used ?

R: On a local level, there is limited budget on health , which is decided by the interest of mayor/deputy-mayor and thus, the programs and plans of local level are not based upon statistics and there is no situation for that to happen.

I: How do you share the data?

R: In the health institution, data trend are pasted upon the wall in whihch we can see target vs achievement but in municipality , we have not done anything like that.

I:Do you have any examples regarding the aspects that have made it easier after implementation of DHIS2?

R: It is very easy in DHIS2 system like timely reporting. A sense of reporting on time has developed among procrastinating friends, so the data comes on time.

I: What motivates you to operate DHIS2?

R: (Laughter) ... I feel like it has to be operated and perform my task. Also, if I don't operate it properly, I feel that my performance will go down which makes me want to give more and prioritise this task over others.

I: What are the main challenges in DHIS2 currently?

R: It is very annoying at times. When we need one thing and are trying to generate one thing, it generates other.

I: What problems are reported by Health institutions?

R: No problems are reported.

I:What is the status of training relating to DHIS2?

R: Personnel in Health post have recieved trained but health personnel of Urban health Clinic and Basic Health Institution are yet to recieve the training.

I: What support do you get from provincial level or higher authority ? Is it enough?

R: Not much support is recieved from district level because they are not updated as well. If I need help with any problem, I make a call to provincial level and they guide me after which the problem gets solved.

I: What can be done to make DHIS2 more effective?

R: We need a software in which we can keep profile of the patient where every details of the patient is available and that record is linked with DHIS2. If this was possible , data quality would be improved as well.

I: How can we increase data utilisation? Do you have any suggestions?

R: We need to give attention to data quality and improve data quality.

I: Are reading materials/manuals regarding DHIS2 available right now or did you get it during training?

R: There is no materials particularly for DHIS2. We did not get manual in the training as well. But we have been trained once, we can learn easily.

I: What are the necessary trainings for upcoming days?

R: .....(Long pause ) .... DHIS2 and LMIS both need to be linked so that logistic management and data management is easier.

I : Interest of people's representatives , admistation and other stakeholders ?

R: All of the individuals at municipal level are not aware. IT Officer sir of municipality has some knowledge of it and he responds well .

I: How do they understand the importance of data?

R: They don't. (Laughs)They don't understand what is data, what it trying to portray they don't know.

1. **IDI with DHIS2FP_Gorkha Municipality**

Gorkha Municipality

Public Health Inspector

Male

Years of experience using DHIS2: 6 Years

I: Since how many years been using DHIS-2? What data have you been entering in it?

R: After I started working in Gorkha Municipality in 2074, I have been using DHIS-2 with the basic training provided. We were not fully recruited as a staff of Gorkha Municipality at the beginning, as we were here with some work purpose. So, we couldn’t fully participate in the training as well. Therefore, I learnt most of it with friends and have been using it since 2074 and using it till now. Until 2077/78 I entered the Data of all 14 health institutions by myself and later after 2078/79 Shrawan, after receiving the training provided on DHIS-2, all health institutions have been reporting the data by themselves and I only enter the Data of local 1 or 2 hospitals.

I: What is the reporting status of Local health facilities since then? Are there any problems?

R: When I first used DHIS2, even though I was not totally familiar with this. I used to fell that data were not well organized, and now I see the same with the staff from health facilities. Even after receiving the training, they are confused about entering the data. They mostly get confused with the tools and matching of the data. Some data also gets cumulative, for e.g data of family planning, Data of Filariasis (Sorry I said filariasis), I meant to say data of Leprosy. So, what actually happened is that, to update as last month’s total patient as 1, data kept on getting cumulative and later it looked like there were 12 patients in a year.

I: How do you address them?

R: Now, we only enter it once and leave it. As entering multiple times showed the multiple patients. In case of family planning, we have to enter it every time but later during the evaluation meeting and all we explain the scenario related of data in how it’s getting cumulative and how the error is seen. Hope it gets improved as our staffs keep on using it.

I: What are other problems related with DHIS-2?

R: Apart from DHIS-2, when we have to enter data at the end of the month, our serves get’s down, and it’s not possible to enter data during the day time, hence we take all the registers at home and try work on it in the night time as the internet gets a little better at that time.

I: What is the status of Infrastructure in the facilities of Gorkha municipality? Are things like Laptop, internet, and Electricity easily available?

R: Yes, in case of Gorkha municipality, we have provided every facilities with Internet and either Laptop or desktop, to enter the data and staffs have been using them. Talking about the internet, there is internet in all health facilities, but it’s not very fast and while entering the data sometimes even after entering all the data, the page get’s blank and we have to reenter the data.

I: HMIS form is given in paper but incase of DHIS-2 it’s all online, are both of them in same format or is it different?

R: Yes, it’s all in same format. For e.g if the form says 9.3, it’s same in DHIS-2. However, there might be missing sometimes.

I: Have you received any complaints regarding the saying that there are no different forms and multiple forms regarding UHC and Health posts as services provided by UHC and Health post are different?

R: No not any yet, more like it feels like it’s easier now with DHIS-2. Before DHIS-2, we used to add and subtract data to make comparison, but now it has been easier. Also, if we get able to keep the data on track properly, we can always track the data and see the record whenever needed.

I: How do you review the entire process? Monthly or quarterly or anything like that?

R: We conduct monthly meetings and discuss as needed. We mostly discuss on 1^st^ and 2^nd^ of every month and check if there is any error from any health facilities and make correction.

I: You conduct meeting by opening DHIS-2?

R: Yes, we open DHIS-2 and show the error to them and ask them to make correction. What we mostly do is, we check for error and if one facility has an error, we don’t check another facility for the similar error. We asked other facilities to check if they have similar error.

I: So, it’s easier to conduct monthly/quarterly meeting because of the discussion related to it?

R: Yes, Very much

I: Now it’s said that you have to use data in decision-making? how is it used?

R: Yes, to some extent. While preparing budget and program and when preparing budget some programs are linked to it. Like In the case of the budget ceiling, when we ask the health section to make a draft, we consider data as a baseline. Also, we saw the prevalence of communicable diseases a bit higher in a community so we recently organized a campaign in 16 different places of 14 different wards based on the data. Similarly, we consider data, to organize a program in other places where the problem is seen. We conduct programs like awareness, Health insurance, etc. Therefore, it has been easier.

I: Do you practice performance appraisal of health facilities and rate them accordingly? What about sharing good practice with other facilities as well?

R: Yes, we practice it. We categorize them, e.g. In the case of immunization. TB, Family planning, etc. we compare which institutions have done a great job. In case of TB, we see where the curable rate is high. Where the coverage is high depending on the coverage 1,2,3,4 etc. We have not exactly managed prizes for the institution performing better, however we make sure to clap and appreciate the facilities performing a great task.

I: Now you can easily get access to data after DHIS-2, Have you found any changes in the quality of it?

R: Yes, have been huge changes but We have not been able to do much. At first, I got the training, and I did what I was capable of. Now 20 of us have been trained. Since they are new, I am also learning with them. Since we are in the learning phase, quality this year seems to be a little fluctuated. But DHIS-2 has improved reporting a lot. That’s why we have allocated a budget again to conduct training for the new staff as well.

I: So, budget have been allocated from the municipality level?

R: Yes, from the municipality level.

I: Are newly elected representatives and administrative interested on the things related to data? How much knowledge do they have?

R: Yes. They are not well aware about the things going on with other sections. But when it comes to data related to health, they get interested to it and are also able to present it with the others. We also, present them the data on monthly, quarterly, semi-annual, annual basis by segregating health facility wise, ward wise, disease wise etc. They are really interested on it.

I: Are there any manuals for health facilities regarding the training materials?

R: Gandaki Pradesh have provided training to all of them.

I: What about the training manuals?

R: Maybe they have received some. There’s a book in the end of DHIS-2 They read it by downloading it. I don’t think separate manual is needed as it’s in the system.

I: You said that you are going to conduct the training on DHIS-2, which area on DHIS-2, do you think is most essential?

R: Basic training is needed at first because, one has been taken the training may take a leave like maternity/paternity leave and since we have to report in within 7^th^ of the first of the month. So, it can get tricky for us. Like previously HMIS 9.3 training provided to all the staffs similarly DHIS2 training should provided to all the staffs. Hence basic training for the health staffs is needed before starting the service.

I: How do the province level get feedbacks?

R: It’s good, they check the system and provide review. We get email from Gandaki Pradesh. They show our errors. We get emails from centers as well. Despite saying what the good aspects were, they highlight our errors which is very good for us. For example, they inquire about the accuracy of the reported data, questioning whether it was recorded correctly or mistakenly; if the death rate appears to be high, they investigate whether the data is genuine or if it was mistakenly entered. We get proper feedback from higher authority as it’s helping us to improve.

I: You are using DHIS-2 this well, you have also received training. What motivated you to use DHIS-2?

R: What motivated most is that, when I need the data, I get easy access to it from DHIS-2, which is very important to me. Rather than flipping a lot and lots of paper, DHIS-2 is online and it’s easier for me. I feel that, if I can address some data in the DHIS, it’s helpful for me in the future as we are all selfish. Second one is the data can be accessed from different level. Similarly it is useful while performance evaluation from province.

I: How do you present the data generated from the other programs conducted by the municipality? Do you record them on DHIS-2?

R: Yes, we add all the data on 9.3. We conduct program based on the tool as well. E.g we conduct TB microscopic camp sputum collection, in the area where essential and where the data can be entered. As the presentation of data is very essential to design a program in near future so we add all the data and prioritize it. In case of some reporting, we design a report differently and send it to the responsible authority.

I: You seem quite experienced in working with DHIS, what suggestions do you like to give to strengthen DHIS-2 in Gandaki province?

R: First, I like to suggest that DHIS-2 system server should build that shows no error and it should run smoothly as it helps us to enter the data easily and in a convenient way.

Second, tools for the program that leads to the cumulative data should be revised. Sections to add the new data generated from the programs besides the national priority programs should also be upgraded.

Third, staffs who conduct a program and prepare a report should be given access to DHIS2. They should be well trained. If the training provided for 3 days, the training will be useful forever. As new staff works for almost 20 years and if three days training managed then the staff will work in his/her tenure. This leads to the proper quality of the data.

There are some data that should be matched, e.g. outcome of TB patients seen in the earlier year is entered in the next year (From earlier Baisakh to next Baisakh) if this is seen in the DHIS-2, and in case we have forgotten to work with the patient, this reminds us to work further by searching the case. Same, is with the iron capsule distribution and Immunization program. Hence, if we get to see the data enter earlier, this can help us to track the case. Therefore, some revision in the tool is needed.

I: How effective do you find it in service delivery?

R: It has a huge influence, As the service provided is seen in the record. Second, it has huge influence in transparency as well as it supports in the social audit. E.g., if patient with fever is not recorded but the Cetamol in stock have already been finished. This might create confusion. So DHIS-2 plays huge role to address such issues.

I: Thank you so much for your time

R: Thank you

1. **IDI with Sahidlakhan RM DHIS2 Focal Person**

Sahidlakhan Rural Municipality

Sr. ANM

Female

Years of experience using DHIS2: 2-3 Years

I: How long have you been using DHIS2 and could you please share your experience in short?

R: I have been using for 2-3 years. I have been employed at palika for approximately 7-8 months, and prior to that, I worked at health post. I started using DHIS2 during my time at the health post.

While sharing the experience with DHIS2, we enter the data. By using the software, we have the ability to retrieve the desired data. However, we encounter challenges when there is a lack of electricity or internet connectivity.

With using it, one we can we can extract data as what we want. And another is we face some problems using it when there is no electricity, no internet.

In recent times, after I joined at Palika, we have to verify the data that reported from all the health facilities. During the process of verifying data from a pivot table, I discovered a discrepancy between the data entered and the data reported on paper. When I inquired about this with the staff at the health facilities, they claimed that the data they entered was accurate.

After the problem became **overwhelming,** we report this problem with higher authorities.

I: What they have said about it?

R: They mentioned that, the system is currently being updated, which is causing certain disruptions.

I: You worked in a Palika, what types of problems do health facilities report to you?

R: We have been using DHIS2 for a long time and we experienced fewer errors in the past. However, recently we have faced different problems like work slowly, difficulties in accessing the system, not working, etc. similarly, we have also observed errors in the data. It could be result of ongoing system update.

I: are there problems with data analysis?

R: we faced the problems during the system update process, otherwise we are extracting data properly from DHIS2.

I: are there any problems associated with data entry only?

R: We haven’t encountered any problems with data entry except for occasional disruptions caused by electricity and internet. Other than that. there are no any problems with data entry.

I: how do you solve problems that reports from health facilities and you faced yourself?

R: If any problems arise, we make an effort to resolve them based on our knowledge. When problems occur at health facilities, we use AnyDesk software to investigate them. If we are unable to solve the problem, if we have confusion, then we contact the province DHIS2 focal person for the solution.

I: Do you get support from province?

R: yes, in case they are outside when we calling, then they will provide us a contact number of another person who can provide the support.

I: Do you get feedback from province and district on DHIS2?

R: Feedback means?

I: Feedbacks on DHIS2 besides your phone calls for assistance?

R: I didn’t receive any information as feedback. If another staff from palika received it, I am not aware of it. In there are any problems, I personally contact them for assistance. We have saved the contact number of provincial DHIS2 focal person.

I: What motivates you too use DHIS2?

R: Before the introduction of DHIS2, there was lots of paperwork involved, requiring extensive manual writing. This process was time consuming and posed challenges related to storage and documentation. Now, with using DHIS2 it has fully become computerized, as a result it has become much simpler and more convenient to manage and analyze data according to our needs. Because of these reasons, it has become easier.

I: have you observed any improvement in the data quality after using DHIS2?

R: ………….. yes……….. health facility staff submit their reports in every 2^nd^ of month. In case, they haven’t submitted on time then we contact them in phone call or message in Facebook messenger group. Also, if they have done mistakes in reports then we inform them their mistakes on messenger group. Similarly, we also share evaluation data through messenger group so that they can correct and improve data.

I: most of says, there is no similarity between paper form and DHIS2 form, is it true?

R: it is similar in recent version. In the previous days, we didn’t have a revised HMIS so there was mismatch. In a recent version it is similar.

I: health post, Basic health unit and urban health unit have complained that, the forms are not as per their services, there are unnecessary forms that we need to complete without data and without service provision, what do you say about it?

R: DHIS2 has included all the indicators for health facilities including hospital. What we are practicing that, we enter the data that produced in our health facilities and for blank forms that doesn’t have data, we have to just click the complete option.

I: currently, palika doesn’t need to enter data?

R: our all the data entered from health facility level.

I: do you provide support or guide them?

R: yes, we do, we look data often, if there are any errors, we inform them for correction and verify. We said them for checking once. However, data entry is done themselves.

I: How DHIS2 make your work easier?

R: our monthly meeting held in 4^th^ of each month and all health facilities needed to complete data entry by 2^nd^ of each month. In case, meeting is not organized as scheduled date and time, due to the emergency situation or meeting postponed for 6^th^, 7^th^, 8^th^ of month. In this this condition, if we need data, we can look from DHIS2.

Moreover, it assists in conducting review meetings (monthly, semi-annually, and annually) by extracting the required data as per need.

I: how many health facilities under the health post?

R: We have total 9b health facilities, including 6 health posts and 3 basic health units.

I: do you evaluate the performance of health facilities using DHIS2?

R: recently we have conducted a semi-annual review meeting. In that, review meeting we ranked the health facilities based on their performance.

I: do you evaluate performance based on data?

R: Yes

I: is there a practice of monitoring and supervision?

R: yes, we do monitoring and supervision as well.

I: What is the situation of data utilization at Palika?

R: yes, data is being utilized. For example, we have an elderly program, which we carry out every three months. We have noticed a rise in the number of individuals with high blood pressure and sugar. In order to tackle these issues, we have made decision to conduct the program at intervals of three months.

I: are there any practices to observe the service coverage, disease trend, and display and disseminate these trends?

R: on the bases of semi-annual review and annual review, we examine the extent of coverage in various areas. For example, we observe immunization program coverage. Similarly, we analyze the top 10 diseases based on data.

I: in your palika, the process of planning, decision making, programming is driven by evidence or influenced by the agenda and interest of elected officials?

R: there is another focal person for planning, decision making, he is responsible for it. In my understanding, they formulate and present plan, programs based on evidence.

I: Based on your experience, is there any example where data shows the gap and program was planned to address that gap in the upcoming fiscal year?

R: I joined the palika at the starting of this fiscal year so I could not answer it.

I: how is the interest of elected representatives on data and DHIS2?

R: we oriented them on revised HMIS. On same orientation we also orient them about DHIS2. Throughout their time in the orientation, they were taking it positively.

I: is there a practice of data display in Palika?

R: we didn’t display in the palika, but in health facilities does.

I: how do you share data with elected representatives, administrative staffs?

R: while sharing data with them, we analyse necessary data and printed it out and explain each data to them. However, we don’t display the data by making a flex at palika.

I: there is no space for reporting about the activities that has been conducted by palika level, how do you report them?

R: we prepare a data sheet in excel for such activities. We have to prepare and submit a monthly progressive report to Palika which includes updates on activities such as the rural ultrasound program, EPI, PHC/ORC, and any new initiatives carried out by the health section. While submitting this progressive report, we also attach excel data that has prepared.

I: it means your reports reached in Palika level only?

R: It went beyond the palika level, if there is any presentation beyond local level otherwise it remained at local level.

It would be beneficial if there is a section in DHIS2 for sharing our program data.

I: When did you receive training?

R: DHIS2 training?

I: Yes

R: 2-3 years ago. I started using DHIS2 after receiving training.

I: what are the needed trainings in DHIS2 coming days?

R: In the era of technology and internet, most of the things are changing or being updated. The skills we acquire through the training may not always be applicable in a changing work environment. Therefore, it is essential to have a refresher training to stay up to date and provide high-quality services.

Many new staffs are joining the job. If they receive a proper training, they can perform their job effectively and confidently.

I: is there a DHIS2 trained staff in each health facilities?

R: some have 2 trained staffs, some have only one trained staff but every health facility has a trained staff.

I: opportunities for DHIS2 coming days?

R:………………………..

I: do you get support from province?

R: …… (laughing)………………

Talking on this, we have been doing, what we have to do. The mistakes are occurring while doing the work. They provide feedbacks time to time through email and phone calls.

Additionally, they provide feedback through email mentioning this this indicators showing the data errors, please check and correct it.

I: are locked data unlocked?

R: yes, they unlocked data set for correction. …………….I forget to mention before……………

I: are there any other challenges in DHIS2?

R: latest challenges I faced and health facilities staffs also shared. Our entered data haven’t seen although we have entered correctly. It may occur due to server or system problem.

I: there are lots of complain about overreporting and under reporting, why it happens, what have you observed while working in palika?

R: overreporting not found much, however health facilities do under reporting. We are verifying data after they have entered in DHIS2 by comparing with talley sheet. Despite this verification process, underreporting still persists.

One event was happened, this was the problem of 1-1.5 years ago. At that time 9.3 PMTCT form included testing, counselling, and results, whereas the DHIS2 form only included testing and results. We entered the data by remembering 9.3 form, assuming negative results. However the data was mistakenly recorded as positive.

we entered data thinking negative but it was entering in positive. It showing high positive cases, later upper level has informed how such high positive cases reported. After informed, we check the data and it was entered in the negative data in positive. As a result, the system displayed a significant number of positive cases. Upon being notified of the unusually high number of positive cases, the higher authorities investigated the matter. After being informed, we reviewed the data and discovered that negative results were incorrectly entered as positive.

I: what suggestions would like to provide to enhance the effectiveness of DHIS2 and increase it utilization?

R: I would suggest to make it offline so that we can work offline and sink when access of internet. It is difficult to use in mobile too.

I: How utilization of data can be improved?

R: ………silence…………

I: Thank you

R: Okay, Thank you

**5. IDI with Health Coordinator of MachhapuchhreRM**

Machhapuchre Rural Municipality

Sr. AHW

Male

Years of experience using DHIS2: 6 years

I: How long has it been since you started using DHIS2?

R: I have been running DHIS2 since its inception in Kaski district.

I: Now what types of data you are entering in DHIS2?

R: We enter all the data from immunizations, registered at health facility, OPD services, ANC and PNC services etc. Similarly, Tuberculosis is now entered through eTB, HIV PMTCT, ICD codes also entered there.

I: What about COVID?

R: Now we do entry the COVID vaccine.

I: What is the condition of your municipality after running DHIS2?

R: With the way DHIS2 works now, it used to be very smooth and it would save and accept easily. But now it is not saved and accepted, if you enter it today, it should show tomorrow, but it does not show, sometimes it is only incomplete, there is a problem.

I: So is this the server related?

R: Yes, it is server related, and also system related.

Earlier, even though it was slow in this server, only the net was slow, the server was slow, but after the entry was completed, after five minutes, a week, two weeks, and a month, it did not show incomplete.

I: Does it only show incomplete or sometimes shows too much data?

R: No, it’s not.

I: Too much data?

R: No there is not showing.

I: Less more?

R: Not showing.

I: Where and what you do if this problem arises?

R: We contact with DHIS2 focal person in the province. In the district, there is a DHIS2 focal person, we asked him first, and if problem is not solved then we ask to focal person of Province.

The problem is that if there is a wrong entry and it's been more than two months and we don't see it, provincial office needs to give access to correct it.

I: System locked itself?

R: Yes, it will definitely lock, after two months. If our data entered wrongly and system is locked, we can't fix it when we do a local review.

We had recently open it few days ago from 25^th^ to 5^th^ in our request to correct the data of Shrawan, bhadra and ashwin months.

I: Does Province ask to correct those mistakes and errors?

R: Yes, they consistently provided feedback through messages and emails regarding our mistakes and errors. Along with the feedback, they shared data, allowing us to correct and address the errors and mistakes accordingly.

I: So, these are some technical issues, there other problems like internet, electricity?

R: Recently in one institution has lacked of net and computer also not given, in that organization staff enter the data from their home. They bring 9.3 at home and enter the data. Else everywhere has access.

I: Are there any problems during this data analysis and data extraction?

R: No, there is no problem.

I: So, every institution enters the data their self?

R: Yes

I: What about the training situations?

R: All of us have taken basic training. And we ask HF staffs, if there are any problems in the monthly review and solve there. And in the conditional budget of the Government of Nepal, some amount has been allocated for the entry of DHIS, the verification of the vaccine of Covid. From there we conduct a review or orientation program related to DHIS, HMIS and work to eliminate confusion regarding it.

I: In the monthly meeting there is also discussion on the DHIS2 as well?

R: Yes, discussion happens. As soon as there is a mistake in 9.3, it shares to each other to see, and bring it to DHIS to correct the mistake according to the mistake and to make the correction in DHIS as per 9.3.

I: Now again, it is said that the form of DHIS2 and 9.3 do not match, what is it?

R: Certainly, in one or two places it is differences, else there is no differences on the form.

I: Does the information missed during data entry?

R: No no, there is only one information missed that is home delivery, there is no place to entry the information of home delivery. If there is place to entry the information in the DHIS2 it would be easy.

I: Is there any options to entry on the DHIS2, regarding the program that the municipality conduct self rather than the government budget?

R: There is no any place and option to enter regarding the local government programs. It should be available as “others” option, but there is no provision. And we report it only at local levels, it do not report through monthly meeting.

I: So it should be there?

R: It should be there; we should get the provision to share with center about how much investment has a local government invested? What programs were conducted and what were the outcomes? That’s is not provisioned.

I: Is there any voices arise for it?

R: We had raised, but concerned authority did not listened.

I: Through DHIS2 data could easily extract, despite of that how DHIS2 help you on your work? How it help you?

R: Data helps us to decide health policies, plans, budgets. We get easy to explain to the local level that our recording by DHIS2 is like this and that the situation of our works is like this, we can show the clear picture of our works while the decision maker asks.

I: What is their interest in this data from the leadership class/group?

R: Not so much interest shows on health-related data. We explained to them, lobbying personally, lobbying on the weakness in the health program. I don't think any local level budgets based on data.

I: How do you advocate with them?

R: When we advocate, for example, home delivery from female community health volunteers at the local level is in this number, we had to do delivery stove program (Sutkeri chulo program) to make zero home delivery, local level had to provide nutritional services for safe delivery, more transportation services had to be arranged. The municipality had to arrange transportation services for those with low financial status who could have home delivery. The province has done an air lifting program, but we also had to put up a program for safe delivery through transportation facilities. If we go and tell them that some money has been allocated, it will be allocated.

I: So the local government has less data literacy?

R: No, they have, now since it is the president and vice-president who looks after the social security program or the coordinator of the social development committee is vice-president, so they have taken a little interest. Now they are interested in why this did not happen in our village, this happened to other municipality, and showed their interest. They also updated through social medias. These factors enhance their interest.

I: How they put the health in the prioritization?

R: Still health is not a priority.

I: Has anything made it easier to run health services?

R: A lot of services are being ease. It used to get lost when keeping records in writing many times, now it is possible to get a report of exactly 5 or 6 years at once, the capacity of the staff has also increased, it is easy.

I: Do you conduct or perform any performance evaluation, rank, give prizes from the programs of health institutions in your municipality?

R: We have a MSS program where we compare with the minimum services for accessing the health institution status, whether it is improving or degrading the status of health organization, the government of Nepal developed the standard, based on that evaluation of health institution is performed. Similarly based on the standards which health worker has play more contribution and roles and performed on the program can also be assessed, in this way both health facility and health workers evaluated.

I: So there is a role of DHIS2?

R: Yes it has important role, evaluation is conducted based on that means based on the data.

I: Is there any improvement on the data quality since/after online data entry?

R: Before the data was not corrected. As of now, the current data seems to be authentic. If you make a mistake in one place, the match will not be played, it will not be saved, after not saving, the person who enters the data will know where the mistake has been made and cannot enter imaginary data.

I: Anyway, after the municipality, district and province will look at it, will there be a check in two three places?

R: Yes. DHIS2 also provides opportunity to see the outcome of activities carried out by health workers using an indicator. Similarly, All the health facilities and local level performance can be observed through an indicator. After doing so, it would be beneficial.

I: What motivates to use DHIS2 or to bring DHIS2 to all health institutions in the municipality?

R: People who can play with data are very interested. It should be compatible with this, but if you search for it, it will be very interesting. Who has previously interest on the data, IT related materials, and they definitely have interest.

I: What are the needs and requirements of training in upcoming days for DHIS2?

R: DHIS2 Training must be given to all health worker. Recently, head of health section, another person or maximum for two people. Why should this training be given, only the in-charge of the health branch can't do the work, let the focal person do the work himself. Those who watch family planning do that, those who watch maternal and newborn health program fill that up, each health staff must know what is inside and what will happen inside DHIS2. The program focal persons do not even make 9.3 and they lack knowledge about the contents of the 9.3 form. In-charge develops and entry on DHIS2. Each official work of the health organization must be known by each health workers, and then only it will be improved and go ahead.

I: Responsive individual should know their own part.

R: Yes everything they should know.

I: Is there any training related materials or training manuals regarding the DHIS2 at health organization?

R: Recently no.

I: You have already mentioned lots of challenges about the DHIS2. Is there any other provincial help and support to solve those issues?

R: There is not much extra support from province, they give us feedback, mentioned the mistakes and asks for error data entry, that’s it.

I: And what about solutions for the other problems besides those?

R: Sometimes some pages of DHIS2 is not visible, 16 17 pages is invisible 19 visible, similarly 14 is not visible 16 can visible. In this situation they fix the issues, only after 2 4 months later. There is no other material or physical support, they do the work only when there is a problem about IT.

I: DHIS 2 will easily provide data in the coming days, but what should be done to increase its usefulness at the local level, sir? To increase data usage or its utilization?

R: First of all, the local level data had to be made acceptable at the local level. If I take the data of our local level, DHIS2 will evaluate with the comparison of central level data. That is not, under our rural municipality the total population is only 22394, based on the population there are not exceeds than 312 under one-year children. But they target provided was 424.

So that if we see the pivot table from DHIS2 dashboard, it will show significantly decrease, it won’t show our progress. And data will say the worker is not working properly. Therefor these things should be considered, local level population-based data also should be recognition.

I: What should be done for the data based planning and decision making process?

R: Central government must be given pressure to local government for the data-based planning and decision processes. If the budgeting, planning, conduction of activities based on the data and making them authentic, central government must pressurized to local level, else they won’t do.

After handover of health to local level, they have understood something about the health. However health staff get salary form the central level, where local level giving salary to teachers and other staff. Until the local level understands that health is our program, they will not allocate money to health. Health should be totally under the central government, else it will not be work effectively.

I: What other suggestions do you have for making something better according to your experience while running DHIS2?

R: While running DHIS2 technical problems are mostly appear else still lost of things need to be mention/addressed on DHIS2. DHIS2 should have the provision of recoding on daily basis electronically, so that the entry can be done at a same day and time.

If today I took the BCG vaccine then the data must be entered at once. After data entry of any places either Lahachowk, Gahachowk or Riwan on the same day of activity performed, monthly data must be located at DHIS2 at same time. We did it for a month, filled the empty tally sheet, then filled it in 9.2, then moved it to 3, and then moved it to DHIS2. Why do this, now we have to make paper less. Not only DHIS2, now has to have electronic recording on the spot.

I: That’s all to ask from my side, thank you for your time sir.

R: ok thank you.

1. **IDI with Pokhara Metropolitan DHIS2FP**

Pokhara Metropolitan

Public Health Inspector

Male

Years of experience using DHIS2: 5 Years

I: How long have you been running DHIS_2 here?

R: I have been using DHIS2 since 2075.

I: Which data are being entered into DHIS2 from the metropolitan currently?

R: Specifically, entry is not done from the metropolitan, the process involves generating data from various sources and then entering it there. In such difficult situations where some health organizations face challenges in data entry, we from the metropolitan, carry out the entry. DHIS-2 concept is about generating data from the various sources, and we, the health facilities, enter it from there.

And a total of 72 health facilities, including private health facilities, have been involved in the data entry process. Since these institutions are being reviewed, one or two may be up or down.

I: Then 72 health institutions will enter by themselves? No need to do anything in the entry from the metropolitan?

R: According to specific rules, the entry is indeed done from there, but there are certain situations where problems may arise. For instance, when there is no internet or when there are human resource constraints, the entry cannot be done from there, and it is carried out form here or another sources.

I: What kind of problems do those 72 organizations that report to you about DHIS2?

R: In DHIS2, there are certain issues that requires manual intervention, and sometimes there can be challenges or obstacles that arise, causing difficulties. Some things are warned about things that should not be warned. And sometimes what happens is that it doesn't open when filling DHIS-2, then it doesn't open at the time of correction or when needed. According to our experience, due to the irregular data entry in the correct time even from our health institution, some problems may arise.

I: What to do in such a situation?

R: At that time, we ask the higher authorities, it will be for a short period, then it will be opened again and it will be done accordingly.

I: Apart from these other problems they have something to say?

R: Problems are sometimes in our analytical part. Sometimes there may be problems in analyzing, in fact this program is DHIS2, that program is very good anyway, it is perfect in itself.

I: When we talked about the health posts, they said that if there are any problems, they will keep us in the metropolitan, what kind of problems come from the health post and how to solve those problems?

R: What they keep is that the data is not opened today, I didn't see that such and such data was filled in, they do report problems like that, but how much of it is visible when we open it from our Master ID and we resolve it, and for some issues we ask the province and solve it.

I: How can feedback be provided to the health facility?

R: That would be our monthly review, semi-annual review, quarterly review and yearly review. Feedbacks are provided through those channels.

I: How can feedback be given for data errors or mistakes?

R: We have a specific program called RDQA dedicated to identifying and addressing data mistake and errors. This program is operated through software, and it is currently functioning and ongoing. But in the case of private health facilities, we do not apply that problem, we do not go, there are some problems in private health facilities, whatever they fill will come.

I: You may also have experienced some problems, how to solve those problems?

R: … Regarding DHIS -2……

I: Yes

R: …. When talking about DHIS2, I also keep things in the province, what should happen if Pokhara metropolitan looks at the data of the health institutions operating under Pokhara metropolis and if we are talking about private ones, Pokhara metropolitan will look at the data of institutions that have been approved from metropolitan only. It would be easy to just look. But what we have now is that we have to see all the data from the Manipal Teaching Hospital to the Western Regional Hospital, so it seems that there is not even a little hierarchy there or if they do not enter the data at the right time, in such case we are not able to pressurize them to make data entry timely from these institutions. These issues are what we are facing at the moment.

I: Any solution from the province at this time?

R: For that, you should look at the institutions within the metropolitan city in a regional way… But the reports of hospitals also go directly and reports of these hospitals also connect with us so that there is a little problem to analyze only our own.

I: And I heard there is a mismatch between forms of DHIS2 and HMIS 9.3, what is the problem sir?

R: This is especially, so now that the patterns of DHIS2 and HMIS are in the process of being reviewed. It may be different in some institutions in the case that the forms that have been reviewed during the review process have not been sent to the health institution or have not reached it. But what we recognize and what we consider final is the software that is in DHIS2, they have to follow the recording forms exits in DHIS2.

I: You said, that there is a problem with the internet, how has the metropolitan played a role in the infrastructure?

R: …. Sometimes there will be problems with the internet. We have the budget to bring all the necessary services for the Internet. There is also a situation of doing. Sometimes they run out of balance, it is a problem for 2-4 days.

I: You have talked about lack of human resources, what are you doing for that?

R: Talking about the human resources, Due to the fact that the human resources are somewhat concentrated in the core city and the administration that have a power to transfer the human resources is very close, it is easy to reach the health workers, so problems have arisen in distant institutions. This is a problem since it is the core city center.

Instead of me giving an answer to that, it is the answer given by the administration officials.

I: What is the arrangement of training from the metropolitan?

R: Training is regularly conducted by us and every health facility has friends who have received this training.

I: How do you report the programs conducted from the metropolitan apart from the regular programs coming from the Conditional Grant or above?

R: Reporting for whom, like our program from here we report to the metropolitan and audit watches.

I: Your contributions to health indicators should also be conveyed to higher authorities?

R: We showed this in our annual review, now here the metropolis has its own annual review, in this section- section wise reports also showed there

And it is the government itself, In particular, it is not necessary to showed and answered that what I have done.

and the another thing is that, Which is a conditional grant, the amount of the conditional grant is spent regardless of whether it is spent and remaining grant will be refunded.

I: Then the health related program data of metropolitan will not be added to DHIS 2 and those data remains limited to you?

R: in DHIS 2 is not the place to report our program

I: You have done some advocacy that there should be a place on DHIS2 to report on the programs you have done?

R: we advocacy in our annual review, There is nothing like that now.

I: How has DHIS-2 made your work easier?

R: It's more secure than convenient to store data or in one place. It can see in one click, otherwise, it is our additional work. previously we have to bring the data to the hard copy anyway, now it comes anyway, it is more work. Pokhara metropolitan is now digitizing the recording of HMIS forms and it is in a trial.

I: Has it also helped to evaluate the performance of health facilities?

R: A minimum service standard is another program for performance evaluation.

I: Does data have any role in minimum service standards?

R:…..

I: Have you found any improvement in data quality after DHIS-2?

R: After DHIS-2, how is the data quality going to be and he gives a little warning and it is done otherwise we have to make a record of the data and entry it only after the record is ready.

I: Data loss when tally like before, there are many entries, has there been any improvement?

R: That's the problem, it has no relation with that problem, to solve that problem, the digital recording system that we have recently tried to implement, After the recording system is in place, the problem will be solved, otherwise it will be entered after all the hard copies are ready. However, there is a warning system when entering, if it doesn't match, it says to look once.

I: Can you also prove that there is an entry in DHIS-2 on Paper?

R: That is seen by a RQDA program.

I: Where is the data being used in the municipality?

R: We make programs according to the programs of Pokhara metropolis according to the diseases that come in the data, but we cannot give an answer based on the central level.

I: When you make the annual work plan and budget?

R: It is a little related to that, in the way that there is a problem in which place, according to the data, for example, in our ward no.32. When we screened, many elephantiasis patients were seen, so we kept the related program in the matters, similarly in senior citizen related programs or in the slum areas issues or if a special disease was detected in any places. We proceed accordingly.

I: Is it also used in the decision-making process?

R: There is some kind of use.

I: You said that it is the local government, how much data is used when implementing and evaluating programs at the local level?

R: Data are used in the health sectors planning but they don’t look at the health data in the whole planning process.

I: How is data literacy towards public administration? How is it that the program should be based on evidence?

R: They do not know that they will get advance health data. We try to share data on annual review but I don't think they are well known about the level of data are available.

I: Where is the other use?

R: if any unexpected new data appears in a place, such as the death of a child or other diseases, our monitoring and supervision should be the process of taking health workers to that place.

I: Do you do onsite coaching in DHIS-2?

R: we do. We have an onsite coaching program. And we do this by calling monthly.

I: It was said that the paper will be discussed in the monthly meeting of the health post.

R: No, it is not always like that, it is discussed quarterly by opening DHIS2.

I: What thing motivates you to run DHIS-2?

R: This is our work, what else motivates us, it was done because of work. No extra allowance. It is easier to use the computer than to sit and write with a pen on paper. Inspiration comes from there too. As for the other motivation, it makes sure that the data is not correct and that the warning system is good.

I: What are the opportunities or scope in DHIS_2 now?

R: DHIS2, I don't see it an advance technology. We need data like how many people in that ward or that community have taken which services, not how many people have been served by this health institution. That data is needed to make a special plan, but in this current system, it is said that this organization has provided services to whom, it does not mean that this organization has provided services. It does not make much sense to take that kind of service.

I: Then the forms of HMIS need to be corrected?

R: Rather than the HMIS form, there is an electronic health recording system data that the hospital uses. The recording system in the entire health facility had to be digital. After digitalization, the address can be analyzed wherever one goes or in the same way. It can be made useful, otherwise if a particular hospital provides so much service now, it will not be very useful later.

I: It is not being discussed in the province or other bodies

R: As a result of the discussion, we now have a trial in three institutions. Now, if it all goes well, we will scale up. We have a target of reaching fifteen institutions in the coming year

I: Do health facilities have training manuals and other instructional resources connected to DHIS 2?

R: Resources are in our DHIS2 system. Not hardcopies but softcopies are available

I: It is also available in Nepali?

R: yes

I: Health facilities reported t not having or not being able to locate those documnets?

R: Their techniques are not enough

I: What kind of training is going to be required for DHIS2 in the near future?

R: Training requirement of DHIS2 that, revised of tools of DHIs 2 that became the need for training could have been implemented immediately as soon as the revised tools became available, training could have been given immediately. One issue is that if the person viewing the program edits while they are sitting here, they will done well and get a good date for their program

I: According to your experience, give your suggestions to make it more effective?

R: As per my experience given DHIS2 is not, we have to develop the software as I mentioned before. Where that person from, then is the data is required. For example, in planning, we need the data of how many people of Ward No. 8 took the first ANC. It is difficult to estimate how many people the health facility has serviced in ward no 8 because people does not just came from only ward 8 , there are more outside. After that, take the service indicator in the population here and joining it. It was a little useful because I could see that there was work left.

I: What can be done to increase Data utilization?

R: to increase Data utilization, that's the exactly I said earlier. In order to increase the utilization, we should have a system/ mechanism to know how many services have been provided by the hospitals and which services have been taken.

I: How can the utilization of the data be increased at the human municipality level?

R: For example, in a certain area or territory if there is more incidence NCD, we could bring the program there. In this way, the program of the NCD could be installed

like, because of the large impact of dialysis, the metropolis itself has focused on establishment of dialysis center after looking at data if you look at it, there are people who bring recommendations for total dialysis from here, Due to the increase in the patient population and then the condition of waiting for the turn, On that basis, we had to decide how much to provide the service of Dialysis, It can be used like that

I: Finally, if there is anything to add to the thread I missed, please put it?

R: No almost all came, so far the best thing about DHIS 2 is this reporting system now we want to connect to the recording system in DHIS2. DHIS2 has also acceped it. so that now recording is also good and let's not talk about Nepal ……..We Pokhara Metropolitian city have to talk about ourselves, each facilities of PMC should have high quality of internet services, then this system is moving forward, it is very good….

Now, we do not have any difficulties due to manpower .But there is a slight deficiency in the IT related works due to senior people are here in metropolitan. However, since new people have also come, we have been adjusting on it in some places the branch itself has done and in some places Health section also do it.

I: Thank you

1. **IDI_DevchuliM_DHIS2FP_Nawalparasi**

Devchuli Municipality

Sr. AHW

Male

Years of experience using DHIS2: 4 Years

I**:** How many years have you been using DHIS tools?

R: Four

I**:** Can you share your experience in short on using the DHIS tools for four years?

R: At first, I found it difficult. But now, when we use the tool, sometimes it does not work. The system does not function properly, and the server hangs when we need to enter data by the 10th of every month. Additionally, the data gets cumulatively added, which is not the intended behavior.

I**:** We have used the 9.3 version for data entry, correct?

R: Yes, that's correct. It's version 9.3.

I**:** Are all the health divisions in this Palika entering the data by themselves?

R: No, only four divisions, including us, are responsible for entering the data ourselves. The Health Post (HP) and City Hospital (NH) have their usernames and passwords, so they enter the data by themselves. We are also responsible for entering the data of Basic Health Care Services (Adharbhut Swatha Sewa)

**I:** How are the conditions of the Health Post (HP) and City Hospital (CH) regarding data entry and reporting?

**R**: The conditions for HP and CH are good. They complete their work on time as they only have to enter data for one section. On the other hand, Nagarpalika has to enter data for twelve sectors, which takes a while.

**I:** How has the DHIS tool influenced your work?

**R**: The DHIS tool has made our work much easier. We no longer have to search for physical files as all the records are entered and stored in the tool. It is convenient to search for trends and patterns over the years. Although I learned to use the system later in my career, I am still able to find the information that is needed efficiently.

**I:** How has the DHIS tool been helping in conducting the monthly meetings, analysis, and review meetings?

**R**: The DHS tool has contributed to conducting these meetings. Instead of relying on hard-copy files, we extract the necessary data from the tool and use it for discussions during meetings. It allows us to have quick access to the required information and facilitates analysis and review.

**I:** However, do we still cross-reference the data entry with the hard copies?

**R**: Yes, we do cross-reference the data entry with the hard copies at times. This is done to identify any potential errors or discrepancies. It helps us ensure the accuracy and reliability of the information.

**I:** How has it helped Nagarpalika in providing health services?

**R**: The DHIS tool has been immensely helpful in this regard. It aids in identifying the coverage set and trends throughout.

**I:** How has the DHIS tool helped in reviewing the performance of the health sectors?

**R**: The DHIS tool has been of great assistance in reviewing the performance of the health sectors. It helps in evaluating whether the entitled personnel are proficient in coding and diagnosis during fieldwork. By analyzing the data recorded in the tool, we can assess the accuracy and quality of the information provided by the health sectors.

**I:** Is there any difference in the quality of data before and after using the DHIS tool?

**R**: Yes, there is a notable difference in the quality of data. Previously, we relied solely on filling out paper forms, which had limitations in terms of accuracy and accessibility. However, with the DHS tool, we have improved data quality through regular discussions about errors, possible mistakes in indicators, and the identification of discrepancies. The tool facilitates the identification of mistakes and allows for timely corrections. Additionally, we now cross-check the data in all the monthly meetings, further enhancing the data's accuracy and reliability.

**I:** Has any of the statistics from the DHIS tool been used by the local government representatives?

**R**: No, the statistics from the DHIS tool have not been utilized by the local government representatives. The data primarily serves the purpose of the health division and us for further planning and decision-making related to health services.

**I:** In terms of health, where has the data or statistics been used?

**R**: The data and statistics have been used in various aspects related to health. They have helped identify the inputs and outcomes of various programs conducted over the years, such as video room delivery and health camps. Additionally, the data has been valuable in the formation of Annual Work Plans and Budget Planning (AWPP), enabling evidence-based decision-making and resource allocation for health-related initiatives.

**I:** Has the DHIS statistics been used in supervision and monitoring?

**R**: No, due to the small size of our Nagarpalika, we do not extensively utilize the tool for supervision and monitoring purposes. However, we are responsive and provide necessary replies whenever there is a need for clarification or reporting related to the statistics.

**I:** What has motivated you to use DHIS?

**R:** I would say my motivation comes from within. As someone working in a position where I need to be knowledgeable and aware of my work, I am self-motivated. The tool allows me to efficiently manage and analyze, which ultimately supports better decision-making and planning in my role.

**I:** Have you encountered any challenges during the use of the DHIS?

**R**: Yes, there have been some challenges. One significant challenge is that there is only one person responsible for DHIS 2 data entry in our Nagarpalika. It would have been beneficial to have additional personnel to assist in identifying errors and filling in the data. In case of incorrect entries, we sometimes need to resort to running wave evaluation techniques, which can be time-consuming.

**I:** Have you faced any difficulties with internet access or electricity?

**R**: Fortunately, we have not encountered significant difficulties in terms of internet access. We have separate access for the Health Division, ensuring a stable connection. In terms of electricity, we have standby generators to mitigate any potential power outages.
**I:** Does the state government or any higher-level authority assist in addressing the challenges faced?

**R**: While I don't encounter difficulties in data entry or minor analysis, for more advanced techniques like pivot tables, we usually discuss and solve them with our staff. However, if we come across any other problems or challenges, the state government does provide assistance and support when needed.

**I:** Apart from the health division, has the data been used by other divisions as well? What are the challenges faced during its dissemination and understanding?

**R**: The data collected through DHIS can certainly be valuable for other divisions as well. However, in our context, the health division is often overlooked and underrated at the state level. As the budget for health comes separately, other divisions tend to prioritize their budgets, creating a lack of coordination and support. Moreover, the health division lacks a separate head, which further hampers effective communication and decision-making. Despite this, we make efforts to include them in our reports and presentations, acknowledging their support to foster understanding and cooperation, and also continue to strive for effective collaboration.

**I:** What opportunities do you see for the future use of DHIS 2 data?

**R**: In the future, one opportunity for utilizing DHIS 2 data more effectively would be to have collaborative supervision sessions where all stakeholders can come together to review and correct any errors or discrepancies in the data. This would facilitate better data quality and accuracy.

Another opportunity lies in addressing the shortcomings in specific service centers, such as the Adharbhut Swastha Sewa Kendra, where the form lacks certain important pages like family planning and social inclusion. Reporting and requesting them to address these issues are crucial to ensure uniformity and completeness in the forms.

Overall, the future use of DHIS 2 data can be enhanced by addressing these challenges, promoting collaboration, and ensuring uniformity in data collection forms for better data integration and analysis.

**I:** Is there any problem with the server?

**R**: Yes, there is a problem with the server. It often does not work properly and causes frequent disruptions. There have been instances where we have only a limited time, such as 10 days, to make corrections or enter data, but the inflexible server adds to the challenges and delays our work.

**I:** When you address the above-mentioned issues to the higher authority, what responses do you receive?

**R**: The higher authority usually requests us to write a formal letter addressing the issues. However, even after submitting the letter, the problems often remain unresolved. The challenges and issues are numerous, making it difficult to capture them all in a single letter. It becomes evident that these issues cannot be effectively resolved solely through written communication. Instead, a collaborative approach where all stakeholders come together for problem identification and resolution would be more effective in addressing the concerns comprehensively

**I:** What is the situation with DHIS training?

**R**: I have personally undergone basic training for DHIS at Gorkha, and the other four members of the health division have also received training. However, there are no plans to organize a training at the Adharbhut Swastha Kendra due to the lack of basic infrastructure such as computers, laptops, and trained personnel. Additionally, Kendra only has two temporary staff members, with no permanent staff. This poses a challenge as there is no guarantee that the temporary staff will continue working after the training, making it difficult to ensure the sustainability of DHIS implementation at the Kendra.

**I:** Would it be beneficial if Palika provides support?

**R**: Yes, it would be immensely beneficial if the Palika extends support. The Palika's assistance and involvement would greatly enhance the implementation and effectiveness of DHIS.

**I:** Is there a need for additional training to be organized?

**R**: Yes, there is a definite need for more comprehensive and in-depth training. The current three-day training provided is quite short and covers only the basics, leaving little room for a thorough understanding and practical application. It would be beneficial if the training program allows participants to not only learn as students but also develop the skills to teach others. This way, we can become proficient in DHIS and help train others in our respective areas.

It's worth mentioning that the availability of training manuals in both Nepali and English on the DHIS2 website is helpful and can support self-learning and reference.

**I:** Do you have any recommendations for Gandaki Pradesh regarding the use of DHIS in the future?

**R**: Yes, I have a few recommendations. Firstly, it would be beneficial to establish uniformity in all the forms used for Adharbhut Swastha Sewa, ensuring that they include necessary and relevant information while excluding unnecessary elements. This would enhance consistency and ease of data collection and analysis.

Secondly, addressing the issue of cumulative addition of data is crucial. It is important to find a solution to prevent the data from getting cumulatively added when it should not be. Furthermore, providing the option to view data for specific periods, (eg for Family Planning) such as only the data of the last month, would greatly enhance data analysis and monitoring.

**I:** Thus, it is evident that providing health data literacy training to all relevant parties at the state level is necessary.

1. **IDI with DHIS2 Focal Person Hupsikot Rural Municipality**

Hupsikot Rural Municipality

Sr. AHW

Male

Years of experience using DHIS2: 4 Years

I: how long have you been using DHIS2?

R: I have been using DHIS-2 since last 4 years. This is the 4^th^ running year.

I: Now what types of data you are entering in DHIS2?

R: since this is 4^th^ year running, at the starting years we have had 3 health facilities and we used to entry all the health facilities data from here. After the establishment of local level, the health facilities also established in all the wards where previously no health facilities. Altogether we have now 7 health facilities under this rural municipality.

I: Do all health facilities enter data themselves or do you enter also?

R: They enter data themselves. In some health facilities, there is a no internet access so we help them with data entry else they do enter data in their own health facilities. We have only four DHIS2 trained staffs (2 in Palika and 2 in health facilities). However, nowadays they are they are connected with networking system and they learn quicky, they do not have trouble like us. Despite the lack of training, they are using it but DHIS2 training is needed here.

We started data entry from all 7 health faculties from this running fiscal year. There are 3 health posts and 4 basic health unit. It is possible to strengthen DHIS2 in the recently established health facilities within the wards by delegating the responsibility of hiring and training personnel for DHIS2 to the health post and ward office of their respective wards.

I: How DHIS2 has made recording and reporting efficient or easy in your Palika?

R: It became extremely simple. If we require any type of data such as monthly, semi-annual, annual, or data from any specific years, we can effortlessly extract it.

It is much easier now, in the middle, due to the untrained staffs we face some problems like no reporting on time, data mistakes. Although even trained individuals can make mistakes, untrained personnel are more likely to do so. Additionally, there is an issue with data entry as the system automatically locks after monthly reporting, preventing us from making corrections until it is unlocked again.

Today I received a email notice informing me about the need to correct data in DHIS2. The locked file has been opened, allowing us to address any errors. We already have noted all the mistakes made and we will correct them in collaboration with the health facility staff within a span of 7 days. Although our errors are not extensive, we are committed to fixing whatever mistakes we have within the given timeframe.

I: Does DHIS2 simplify conducting monthly, quarterly, and annual review meetings?

R: We have a monthly review meeting and we extract all the needed information from DHIS2.

I: What is the situation of Data utilization in your Palike while in the decision-making process, planning, monitoring, evaluation, etc.?

R: We gave a printer in last fiscal year and this fiscal year we are providing desktop to all the health facilities with aim to assist all these things. In addition, we are looking to provide internet access and the necessary equipment in health facilities where there is currently no access, taking into account DHIS2.

Although the rural municipality is accessible due to it located in Terai District. Its sources of income are limited because it is situated slightly inland and relies solely on revenue generated from activities related to the river.

Although we receive excellent support from Palika, our ability to deliver services as anticipated is hindered by economic constraints. However, we have devised a plan to address this issue by offering printers and desktop computers within the current fiscal year. This will enable users to enter data using mobile data, even in the absence of internet connectivity.

I: Does the local level use data when planning, making decisions, and program development?

R: our all the actions are driven by data. For example, prior to our yearly meeting at Ashar 10 of each year, we review the data from DHIS2 to determine which programs have had low or high level of achievement and which programs require more attention in the next fiscal year. We utilize this information to request budget for next fiscal year.

I: Is there any example of programs of planned implement, that municipality had done from evidence based data?

P: In health sector here are many programs but, in case of DHIS-2 in last FY, we gave DHIS-2 to health facility for strengthening. Because for data we can say, DHIS-2 is essential.

There are many health related subject matter but DHIS-2 is related to training. Why we didn’t felt the need of training? As training is conducted though health institution we didn’t prioritize it. If we can fulfil equipment, district health institution can help to conduct it.

There is not any objection from municipality, if we need any things regard to health sector. In case of other municipality, there may be problem related to budget but in our municipality there are not such problems.

I: is there any improvement in data quality after using DHIS-2?

P: We are operating DHIS-2 immediately after it has been started. In the beginning days, some matches were not seen but continuous improvement are seen in recent days. And another things is that, I am working here these days. And there will be still availability of registered data even though if I will be transformed. Registered data can be misplaced but from DHIS-2, everyone can have access to see the data which is the major positive aspect of DHIS-2.

The data I have entered will remain here even though I will get transferred. It takes a lot of time to search the registered data from manual file. But in case of DHIS-2 we can get any kind of data with in 30min. If there will be feature of entering data about the program that have we been conducted from other municipality Eg: camp conducted from other municipality, then it will be more appropriate.

I: What are your experiences in underreporting and over reporting cases?

P: Here is no such problem.

I: Does it have facilitated in health services after the use of DHIS-2?

P: YEAH! Absolutely it had made easy because, when we are conducting monthly review meetings we displace the data. We can also monitor the staff and show them, their mistakes and suggest them for improvements. As an example, if we talk about immunization then, we are in stage to achieve full immunization. For this we conduct meetings and analyze the targets and achievements. If they didn’t achieve the targets then we discuss about the reason behind it. We overview their entry and target population and analyze and discuss achievements.

Example, Children who have received BCG vaccine have received where and what other vaccines?

If we see the data from earlier time then, 7 children received BCG vaccine but children receiving other vaccine after BCG has declined to 4. Then among 7, where does 3 other have received the vaccine? If this things were recorded in registration form (manually) besides DHIS-2 then we have to search it, which will take a lot of time. Now it had made easy to show it from DHIS-2, which have helped us in monthly reviews also.

DHIS-2 had made us easier and as a whole to the nation. The data we enter here can be immediately seen by them. Before this, we get feedback from higher level. Sometimes, by mistake we enter 7 case of Kalazar, without even single case of it. Now monthly data are monitor from district and they highlighted overall mistakes. Then we will see and then we correct it in time.

I: DHIS-2 makes easier for monitoring, down oftenly you will do monitoring and supervision?

P: We have 2 institution far away. And they also come for monthly reviews meetings. Rather than doing supervision and monitoring, it is easier to point mistakes of data in DHIS-2 and monitoring it from register. For example, in nutrition new growth monitoring is exactly seen. BCG injected children are compulsory and if there is not availability of that then what is the matching condition of nutrition and immunization registration?

So, because of this it, it had made us very easy.

I: In your local level, through viewing data, health facilities’ performance evaluation is done through performance comparison?

P: We have a one problem, as this local municipality is back warded/ rural. We also call this palika as three floats. In upper part, it is back warded or critical and in middle part it is hilly and we also have terai. In earlier time we used to have 3 V.D.C and after the compilation of 3 VDC now we have one Palika. We have controversy regarding ward’s boarders, we have ward number 1 across the river also. Target population doesn’t come according to the ward. As we are ward number 2, so we cannot say about how much we have achieved. Next we have ward number 6 which is placed separately. It is easy form them to estimate targets and achievements. But we have controversy in population distribution and wards so it is bit difficult for us.

Now Gandaki Province is planning to make health profiles and after making profile we will know about the population distribution in each. Then it will be easy for us.

I: There is practices of sharing best performer of HF declaration and learning and best practices from those health institution?

P: We are not doing this in our time. Because from our district, Bulingtar RM is ranked as district first and 10^th^ position from Gandaki province. If we will do this practice then we can go up to health institutions.

We are ranked as 5^th^ according to district and 49^th^ position as Gandaki province wise. As closer we have come, more achievement we had made.

Now president of our RM has asked about why we have not achieved as ranked number 1. This year we have targeted to achieve 3^rd^ position from 5^th^ position.

I: What motivates you to use DHIS-2?

P: This work is obvious for us. After training it is easy to use and data are needed to us also.

Now I don’t have to operate it. In earlier time, I was alone in section and I have to do from 3 different health facilities. Continuously, health facilities staff are provided training and now they enter data by themselves. So, I don’t have to do all.

I: You have mentioned some challenges, beside that what other challenges has been mentioned by your friends (stat) or that you felt?

P: When we talk about challenges, firstly we don’t have training. If training is provided then we can tackle the challenges. They don’t know how to operate data, analyze data, make charts etc. They don’t have enough data so they enter data only in one-way basis.

We give them basic training for general work but it will get better when concerned authorities will give them better training. If it continues and concerned authorities don’t give training then we have to do training ourselves. But somehow if training will be provided for 2-1 person annually then it will be easier.

It is learning things. After continuous learning we will get to use it easily. Somehow, we are also in learning phase.

I: During data entry process, is there anything you find difficult?

P: There is no difficulty during data entry. But what is difficult for us is, to upload data and to evaluate/ analyze the progress of programs. We are not excellent in these factors.

We can do many things such as data operation, entry and viewing data but we are poor in data comparisons and data visualization. So, we must learn these things.

I: We have internet problems?

P: NO! What problem we have regarding internet is, we have internet manager/ provider. As we are somehow rural so sometimes problem are seen for little longer time. Beside it we don’t have other issues but we have internet problem in periphery area of other institutions.

I: You have problems such as data lost due to technical issues?

P: We don’t have data lost problem but mistakes during data entry is different thing. One data lost problem was seen in ward 3. Problem was seen because they have entered data in data entry sample practice. So due to which the entered data was only viewed in his but not here. In this subject, one expert staff/ sir had come here. He viewed the problem and consulted with higher level staff but problem was not solved. After that he returned back, I have viewed the problem. Data entry was not done in appropriate place as it was entered in sample. So, they again entered data.

I: To solve these challenges what initiatives has been made from federal or provincial level?

P: Some facilitation is done from province level. In last year, one of staff got training but training was not conducted this year.

But what is seen in training is, they receive training and get transferred. But now as we have local level, and all staff are from local level. So, if one will get training then it will be easier.

I: Any feedback provision is seen from province?

P: feedback are usually given. Yesterday also they have sent email. Usually these things happens. They make us realize our mistakes.

I: To use DHIS-2, is there any training manuals or learning materials?

P: YES! We have manuals

I: In upcoming days, what possible opportunities can be made to make DHIS-2 more reliable and good?

P: opportunities are; all institutions enter data from their respective health institutions. And data from them remain for so long times. Therefore it is easy also.

To operate and report data it is so easy. For example, if you will ask some data, we can provide it on the spot. But if it should be given without use then, you have to wait for 1-2 days.

I: To make DHIS-2 more effective in Gandaki province, what appropriate suggestions you want to give?

P: I am using DHIS-2 since 4 years. We conduct different activities like eye camps, VIA camps and many other camps. But these information cannot get added in DHIS-2. These activities will be added manually in files but we will forget about the place we have kept. We also keep these things by making separate folders but it will be easy if we will have option for extra activities in DHIS-2.

For example, we had conducted a grant camp in Magh month. Many people were benefited. If DHIS-2 will have option to keep such activities and all activities from local level then, we can save it. It will also be easy to search those data in later days and for others to view our work.

I: Lastly, is there anything that I have forget to ask or you want add further?

P: Nearby us we have Ayurveda section. If the service provided by them can be seen by us through DHIS-2 as a separate portal then it will be more effective.

Now in recent time, Ayurved section report to district but we don’t know where district reports their data.

I: DHIS-2, LMIS and e-TB are effective separately or they should be complied in one?

P: Yeah! It will be easy if these data will operated singly. Our friends have load with works also. During this peak time with work, if e-TB, DHIS-2 can be entered separately it will be superb. But due to same nature of work, if all will be included in same portal then it will be easier.

1. **IDI_DelomaRM_DHIS2FP_Mustang**

Delima Rural Municipality

Nursing Staff

Female

Years of Experience using DHIS2: 7-8 month

I: How long have you been using DHIS2?

R: I have learned operate DHIS2 at the beginning of this fiscal year i.e., Shrawan Bhadra and after two months, I received training on it.

I: Currently, which data have you been entering in DHIS2?

R: Currently, we are entering the data that are reported from health facilities.

I: Do you enter COVID data also?

R: We only enter COVID data when there is vaccination program, currently there is no vaccination program.

I: How many health facilities have using DHIS2 at your palika?

R: In the Palika, there are a total of five health facilities (3 health posts and 2 CHU). Due to issues with internet and other challenges, we were entering data from Palika ourselves. However, for the past two months, we have assigned responsibility of data entry to two health posts.

I: So, 2 health post enter their data and remaining facilities data entered from Palika?

R: umm….. we assigned health post a month ago. Usually, we enter data ourselves.

I: Are the necessary supporting equipment’s for using DHIS2 available in health posts?

R: No, there are no all-supporting equipment’s available in the health posts. We just have a laptop for entry. We have plan to strengthen infrastructure to all the health facilities in the next fiscal year.

I: Currently, they entered their data from Palika laptop?

R: Yes, last time we did entry this way, but this time we have equipped two health posts with laptops. Next three health facilities will be provided next fiscal year.

I: Besides internet problem, what other problems do you face when using DHIS2?

R: In the DHIS2?

I: Yes, while DHIS2 Operation?

R: umm….. its not exactly like that, but the internet is crucial. In addition, when the internet connection is lost while entering data, sometimes the entered data is saved and sometimes it isn’t. Similarly, the completed data are also not displayed.

Otherwise, it is good and its good for me. But sometime software doesn’t work for 1 to 2 days. These are the major problems, otherwise I am satisfied.

I: Are there any problems particularly with data entry?

R: There is no problem with data entry, problems arise when internet disconnected. Moreover, there are instances where data is entered correctly but does not display after 2-3 days. In such cases we re-enter the data. I don’t understand why this happens. It could be due to internet issue or other any issues. Another concern is the need to verify the accuracy of the data after a couple of days.

I: Problems when data analysis?

R: I enter data only, so I don’t have much idea about it. But I heard that, it shows more data than the actual data entered.

I: What about the situation of training?

R: We haven’t received any training; we are using it by learning ourselves from various informal sources.

I: You also didn’t receive training?

R: No, we have not received any training thus far. Even our health coordinator, who has been using DHS2 for two years, has not received any training. None of us have been trained.

I: How do you solve these problems?

R: First we check data either data entered or not, if data is not entered than we will re-enter it.

I: Do you receive support from province in case of any problem?

R: We still haven't received the support we need, but we're attempting to troubleshoot on our own to see if we can resolve the issue. If our efforts are unsuccessful, we will seek assistance from the District Health Office.

I: Are their trained staff in district?

R: They assist us to solve problems, I think they have received training.

I: Monitoring and supervision from District and province?

R: I am not aware about monitoring and supervision from province. It is possible that they do not visit us because of the distance between our location and the province. However, the district focal person consistently visits us, communicates via email to inform us about any data mistakes or errors, and frequently contacts us through phone calls. The focal person in the district is so good. They do their work seriously. We do mistakes sometimes but coordination from district DHIS2 focal person is good.

I: Orientation of revised HMIS forms?

R: Yes, we already received orientation on revised HMIS.

I: Mismatch between HMIS 9.3 forms and DHIS2 forms?

R: Most of the forms are similar but in some forms are not matched properly. However, the data are not lost. There are spaces for data entry but we have to do some exercises. For example, in the HMIS form, there is column of total old OPD cases and total new OPD cases but in DHIS2, we only enter new OPD cases, there is no space for old cases to entry. However, it comes in the first page of total beneficiary’s section.

I: How DHIS2 made your work easy?

R: DHIS2 simplifies the process of inputting health data by allowing users to easily access the software. Despite occasional issues, entering data is straightforward, as all health program information can be conveniently inputted into designated sections. I appreciate this feature and consider DHIS2 to be a commendable software among others.

I: Is the process of recording and reporting, data analysis simplified?

R: Yes, it makes easy. However, users’ express dissatisfaction as it fails to display precise information. On occasions, it presents more data than what was initially entered.

I: Do you correct data in this case?

R: No, we present whatever the software shows, we don’t do any data verification. If there is anything comment we will ask seniors and will do correction.

I: support in meeting conduction?

R: Yes, it does

I: how?

R: because it shows all the data in a time. I don’t know much about it. Other expert staff generate data from DHIS2.

I: Support in providing quality health services?

R: The updated HMIS incorporates distinct registers for various programs such as NCD and reproductive health, facilitating the segregation of data. This separation should be helping to people working in the decision-making level of upper level.

I: Support in performance evaluation?

R: We do not separately by Palika. District level perform the performance evaluation of health facilities, when doing this by district level, we should compare performance with other palikas.

I: Any improvement in data quality?

R: I don’t know, what is the situation, but after using DHIS2 we can perform the data analysis in a short time.

I: Utilization of data at palika in decision making, planning?

R: It made easy to data analysis. We can present the data with elected representatives on the situation of different programs like percentages of immunized children. We can extract ward wise data also.

Everything runs with the data; we review the data of each program (ward wise data, 3 years trend of data) and identify the gaps and we work accordingly.

I: Data literacy among stakeholders?

R: It is good.

I: You get proper support from Palika?

R: Yes

I: Motivation to use DHIS2?

R: Firstly, it’s our duty. And another I enjoy using DHIS2 instead of paper-based data analysis.

Similarly, it is easy for data entry; HMIS forms are prepared according to DHIS2 form despite of some mismatch.

I: Data sharing practices?

R: There are no such practices. We don’t display data by preparing flex. In case district need data and demand, we provide necessary information to them and welcome their feedbacks in case of any errors or mistakes identified.

I: Needed training in DHIS2 in coming days?

R: Need both basic and refresher training because we don’t get any of them. The trainings are running in other districts but in our district, there is no training regarding DHIS2, it could be due to low seats in the districts.

I: Suggestions for betterments?

R: Firstly, training must be provided to all the staffs then it should easy to operate. In addition to this, it would be better if the system work offline and save the data when internet disconnected.

I: What is the situation of electricity?

R: There is electricity. Also, there is a solar for backup.

I: Situation of internet?

R: Internet is available in 2-3 health facilities. Mobile data also work properly. Sometimes there is no network problem for 2-3 days at that time it doesn’t work.
