# Supplementary material for: Health professionals’ experience on District Health Information System (DHIS2) and its utilization at local levels in Gandaki province, Nepal: A qualitative study
Source: PLOS Glob Public Health. 2024 Mar 27;4(3):e0002890. doi: 10.1371/journal.pgph.0002890 (PMC10971587; doi:10.1371/journal.pgph.0002890)
Supplement: S5 Text — (DOCX) [file pgph.0002890.s006.docx]

**In-depth Interview Transcripts – Health Post level DHIS2 Focal Person**

1. **IDI with DHIS2 Focal person Fedikhola_HP**

Fedikhola Rural Municipality

ANM

Female

Years of experience using DHIS2: 3 Years

I: In depth interview with health post DHIS-2 user. Shall we start our interview?

R: Yes

I: As you have mentioned, it’s been 3 years since you have started entering data in DHIS-2. Please share your experience regarding what types of data you enter, your role in DHIS-2 and types of activity you do with DHIS-2?

R: Yes, it’s been more than 3 years since I am entering data in DHIS-2. As it is health post so we entered monthly data. We entered data regarding reporting status of immunization, family-planning, safe motherhood, OPD service. Firstly, all the services we gave are reported in a report and finally entered in DHIS-2.

I: In earlier time all the report should have to submit in municipality. I guess in recent time also, you should have to submit the manual written report and DHIS-2 side by side. And how helpful it has been in recording and reporting after the use of DHIS-2?

R: In my opinion, we are not able to use it regularly like we used to report manually. But in reviews like, monthly and biannually review we use it for analyzing data. And in analyzing the situation in some cases also we do use it.

I: And how has it made reporting/recording of data fast and easier?

R: Most of the times, we are not able to enter the data due to error in server. Mostly whenever we try to enter the data, the server is found to be down.

I: How easy it is and how is the quality of the data from the monthly and quarterly meetings whenever you try to retrieve them?

R: We retrieve the data from the pivot table. Whenever we see error in the data surfacely, we have difficulty in finding the respective errors. In earlier time, error were found in municipality monthly data recording, were data were mismatched and we were not able to find the errors in time. I think it is due to low practice of entering data. We do not use pivot regularly so, we found it difficult using it sometime.

I: And you use DHIS-2 to evaluation program and to see target vs achievements?

R: In earlier time we have 1 and 2 and there was aggregation of 1, 2 targets so it was unmatched. Now we have separated 1 and 2 so we can see it. How target and achievements has occurred and is has achieved or not can be find out. So, we can say we use it.

I: I guess, computer health units, community health facilitators are available here. Do you include their data and enter them?

R: In earlier time Phadikhola health post 1 and 2 was monitored by 1. 2 include basic health center which was not added and entered so it seems like we didn’t achieve the target. Now we enter data of 1 and 2 separately in DHIS-2. As, 1 and 2 has separate targets and entering them separately has been little bit easy this time.

I: So you enter data of here only?

R: Yes, we enter data of here only. Recently we had separated the role also so, we enter the data of here only. Basic health center was also available in 2 but FCHVs were internally from here. As we had 2 health facilities where some roles had to be fulfilled from here and some role had to fulfilled from 2 in earlier time.

I: Do you set target in banners for monthly and annual activities?

R: For reviews it has been extracted from DHIS-2. But I am not confident like I do from manually as I found it unreliable. Sometimes by mistakes data can get error so we do manually most of the time. im

I: So, even though you enter data in DHIS-2 but for work you do it manually?

R: Yeah! And later, DHIS-2 data will be re-checked from municipality. If any error occurred then they will correct it.

I: As I have mentioned earlier, for work inheritance also data is needed. For programs you use DHIS-2 or not?

R: Particular for other work we do not use DHIS-2

I: Now in DHIS-2, quality check and data validation can be checked and during entry error can also get minimized so does it help in producing quality data?

R: When using validation, some data were not accepted in earlier time. As, an example; in safe mother hood, first time pregnancy tested by 4 women and in same month only two of them have visited according to protocol then for validation system will show that if 4 women are at first visit for pregnancy then system will do show that there should not be less than 4 women. At same month there cannot be 9month and 4 month. So, for validation such data will appear but it get completed in recent time. And in earlier time, about three year back there was not such conditions but in recent time it shows validation check.

I: If any problem is found for validation check in DHIS-2 then where and with whom you consult?

R: We can consult with health directorate Pokhara Sir’s. Now in recent time we are not facing such problems also so, we do it ourselves.

I: If any problem is found further or for difficulty then who will help you?

R: Firstly as we have health sector for consultation. And we can also consult with health organization syangja.

I: And for supervision also work is carried out?

R: Yes! We do have supervision by health section.

I: And is there any use of DHIS-2 data by health section besides meetings like; for request in resources?

R: Up to this time we hadn’t used DHIS-2 besides meetings. But we did have program like “Meric Mobile” here which had facilities like coding and registration of pregnant women by FCHVs. And we did have compared that data with DHIS-2 but for public work but for planning or for programs we haven’t used. As we have report here but we do not print and use it but we do manually.

I: As you do both manually and through DHIS-2. As you operate data and submit to municipality and I guess municipality work on the basis of data. And does DHIS-2 have affected or helped in service provision or in health services?

R: I don’t think it has affected in any cases. And like immediately reporting of cases like TB case or COVID vaccination it is more fast and easy and can be updated fast then from doing manually. And I guess it does not have affected in negative sense.

I: Besides disadvantages, how it had helped or supported in health service provision like online reporting or for asking resources?

R: I don’t think it had helped in any specific things till now.

I: What do you think about how municipality had used DHIS-2?

R: We are sure that, they must have used it properly.

I: Let’s talk about challenges and opportunities regarding the use of DHIS-2. What are the motivational factors for you to use DHIS-2?

R: For me? I think it is something new to learn and I have learned new skills and also can get updated. Yeah, that’s it.

I: It is from personal view point and if we see it in professional way then?

R: In professional way, if I am far away from my workplace and if I don’t have reports also, I can view data and mobilize it. I guess it is easy access from every part of country.

I: And what are the challenges in using DHIS-2?

R: All the required materials should be available in every health facilities. We do have materials but every health facilities don’t have enough required materials. And there must be facilities of internet access. And trained and skilled manpower are needed. Yeah, these are the challenges.

I: And is there any support from Palikas and provinces to use DHIS-2?

R: I have received training to use it.

I: If you find difficulty in obtaining some data then?

R: Yeah! We do have support for such issues. And if I am busy in some training or other works then they do help by entering data. And if any problem raised also they do support for it.

I: And is there any technical problems?

R: Yes we have technical problems like; internet is slow and we have to use our personal laptops. If we were given laptop then we can operate data anytime. Usually we don’t need laptop in health post for any other works. But sometime due to slow internet and due to damage in materials we can have problems.

I: And during entry time what types of problems you face?

R: Some time due to server error and sometime due to lock of data. And sometime due to busy schedule of staff and not being able to update properly.

I: And there are boxes for data entry is it a problem?

R: Yeah! Sometime it is time consuming. But it doesn’t takes that much time. I guess 30 minutes is enough and I personally feel that tabs are time consuming.

I: Do you personally feel problem during using pivot, dashboard, and exporting data?

R: Its OKAY. I do not have problems in such things.

I: As you have mentioned that you use your personal laptops for entry. Is there any other problems and how you are facing and tackling those problems?

R: Yeah we use from mobile data if there is no WIFI provision. I want to share my recent experience, “We usually submit and finish data entry by 15^th^ of every month, I was so busy in trainings so we did completed our entry by 7^th^ by doing it in early time.

I: As we have moved to digital era, what can be the possible opportunities of using DHIS-2 from your point of view to make it better?

R: It is totally paperless work. Anyone can operate and use it from every part of country. We can also evaluate our progress by analysis. As it is less time consuming than manually. We should be able to use it properly.

I: Is it prioritize by municipality?

R: Yeah it is in priority list. More like DHIS-2 review, it is prioritize for reviews. Municipality staff monitor and support in solving and analyzing errors.

I: How many trainings and workshops have you been participated?

R: I have involved in only one training session of DHIS.

I: Along with training did you get training manual and review materials?

R: We did received HMIS book but we don’t have DHIS manual. I don’t know we have manual or not but I didn’t received any of them.

I: We don’t have manual in this health facilitate?

R: No, we don’t have any.

I: Many new features are adding in DHIS-2. And as you have said, you only have received only one training so, what can be the possible need of trainings regarding with DHIS-2.

R: If new features will be added then

I: Is there anything you wish to get added in training so that data analysis will get easy.

R: It’s like if we don’t use continuously then we forget it. I think update training is required as a revision. If we will enter data once in quarterly or annually basis then we will even forget the starting phase of entry. If we’ll have manual then somehow problem will be solved otherwise we’ll forget it.

I: As you have mentioned DHIS-2 is not in use in other health post and to make DHIS-2 more effective in use, what can be the better suggestion for appropriate and oriented use of DHIS-2?

R: Regular supervision and monitoring is required. Should analyze where, how, who and when they are using. And monitoring is required for health post and DHIS-2 user. And it should be prioritize and access in every health institutions. Health credits should be guide and command for the appropriate use of DHIS-2 as a reference. If these things will be applied then it will work.

I: And for overall improvement and effective use of DHIS-2 in whole Gandaki province what suggestions you want to give them as you work and coordinate with municipality and other institutions?

What feedback you want to give them in technical issues, trainings or any other aspects?

R: Yeah, I have already mentioned that there should be appropriate supervision, monitoring of data. And in time evaluation of problem and resolving them in coordination can help for better and sustainable use of DHIS-2.

I: OKAY. These are the things to ask from my side. I guess interview is over for now. Thank you!

R: THANK YOU!

1. **IDI with Health Post In-charge Taksar**

Putalibajar Municipality

Sr. AHW

Male

I: Can you please share your experiences regarding Dhis2?

R: We are using it since 2077-2078. I have been entering data after receiving 2 days training. Besides data entry I have not learned anything like analysis.

I: Challenges during data entry

R: There are no such challenges while entering data but there is an internet problem so that entry cannot be done on time.

I: Now, are you doing recording reporting through DHIS2?

R: Yes, we are doing recording and reporting in DHIS2.

I: Reporting made easy with Dhis2?

R: Yes, reporting through Dhis2 has become easy but due to internet problems it has become a bit uncomfortable.

I: You said that, you do data entry only, do you analyze data sometimes?

R: Data analysis skill has not been developed. Friends those have that skills do it but the skill of analyzing monthly and annual data has not been developed.

I: How do you analyze the data when data are required?

R: When the data is needed, either I come to the municipality office and analyze data with help of sirs, or else I extract it manually from the master copy. But sometimes, if there is no condition for the municipality to come, I ask for help by phone, and I do what he says and extract the data.

I: Do you see the service coverage or target vs achievement using DHIS2?

R: Yes, we do it regularly however the migration has made challenge to achieve target.

I: How much is Dhis2 data used in decision making or problem solving?

R: Monthly meeting are conducted here regularly and problem seen there and their solutions are discussed. But in field level, the scenario is different example they are told to look for and bring children who have missed vaccinations, but due to migration, no children are found in the village.

I: Are there any data utilization practices?

R: We do not have any data utilization practices.

I: How much support do you get to run dhis2 from the municipality level or higher bodies?

R: We received good support from local level, we discuss issues in monthly meeting or when we have problems, we also ask for help through the phone.

I: What advice do you suggest to make DHIS2 more effective?

R: The state should establish a reliable network and also provide training to the health workers.

I: What motivates you to operate DHIS2?

R: It is very easy and quick to operate. And when the government is moving towards digitalization, we did not go the opposite way, that's why we are motivated to do it.

I: In your experience, how much data is used in the planning of programs at the municipal level or in the decision-making process?

R: ln local level, health is not in priority. As this is a technical line, the members of the judiciary do not know many things about it. If you are able to coach them well then, they understand otherwise health sector problems at local level are increasing.

I: What challenges have you faced while operating DHIS2?

R: There are no resources, I have to ask from ward office to use the internet.

I: What advice would you suggest to make Dhis2 more effective?

R: In the days to come, everything here should be paperless. For that, it would be more effective if patients could be registered on the computer, medical records should be kept there, medicines should be kept there, registration numbers would also be given from there, and training could be offered to everyone.

Currently, we give a registration card to patient when receiving services but when they come back for service, there is no registration card, so it is difficult to know that they have taken the previous service. If we start looking at our registration register, it takes a lot of time, so it would be great if it could be computerized.

I: There is a discussion feature in DHIS2, how much do you use it?

R: It is not used much because no one reply there.

I: What are the necessary trainings to run dhis2 in the coming days?

R: Data analysis training should be conducted so that annual monthly quarterly data can be extracted and tables, charts can be made. This training should be conducted for two or three days according to how many days are suitable.

The current new friends are skillful, but those of us who are working before could not work without training and resources, so there should be provision of resources along with training. If this is done, we can work easily. Regular monitoring and evaluation is not done by the province, sometimes it is done by the local level, but that is not regular.

1. **IDI with DHIS2 Focal Person Bunkot Health Post**

Sahidlakhan Rural Municipality

Post: Senior AHW (Health Coordinator)

Male

I: How long have you been using DHIS-2?

R: I have been using DHIS-2 for the past 2 years.

I: Did you start using it with or without receiving the training?

R: Used 1 year without the training and the next 1 year after receiving the training

I: What kinds of data are you entering into DHIS-2 recently?

R: Enter all the data of the monthly report at the health post level such as OPD and family planning including all the data in 9.3

I: What is the condition of DHIS-2 in your institution?

R: Good. Till now there is no such problem however reporting around 2, 3 and 4 of every month, the system gets busy. During that time problem of the system unreachable appears. There are no such other problems in DHIS-2

I: Any other challenges besides this?

R: Sometimes the entered data appears and sometimes the entered data gets lost also.

I: Besides this, how is the condition of the internet, computer or other necessary infrastructures?

R: As there is the facility of internet and desktop in each health institution of our rural municipality, basically there is no problem using DHIS-2.

I: Have you been facing problems with analysis?

R: Faced problem with analysis before receiving training but after receiving training there is no problem even with the analysis.

I: Are the problems you mentioned similar or quite different from those that your friends discuss with you?

R: The problems of every friend are almost similar as in health institutions of our Sahid Lakhan Rural Municipality, 50-60% of health professionals have received training. As far as I know, this is the first RM in Gorkha District to conduct training on DHIS-2 and eLMIS. Due to this number of friends wanting to receive training in our municipality is quite high.

I: There has been a complaint that the recording forms in DHIS-2 and the recording forms in the paper are not similar. What is your experience regarding this?

R: There have been a few ups and downs in the OPD diagnosis in the old 9.3 paper and in the OPD diagnosis of DHIS-2 just as the things that were in DHIS-2 were not in the paper and the things that were in the paper were not in DHIS-2. But lately DHIS-2 has been updated and after that there is a similarity between DHIS-2 and 9.3 paper

I: After DHIS-2 has it been somewhat easy in recording and reporting or the paper-based system was easy?

R: Obviously the recording and reporting in DHIS-2 is quite easy than paper-based reporting. DHIS-2 is very easy. Instead of turning the data of 12 months of monthly review meetings and yearly review meetings, the pivot table gives the data in a very short time. Sometimes the data shown by the pivot table and data in our tally sheet mismatches or else it is very easy. It is easy on digital devices.

I: If this problem appears in DHIS-2 where and to whom do you say?

R: We received training from the provincial health directorate, DHIS-2 focal person Sir. There are two competent DHIS-2 focal persons in our municipality. We place our problems with them and if it didn’t work, we personally place the problem with the provincial health directorate and he gives a response regarding that.

I: Is there a practice of giving feedback to you from time to time?

R: There is a practice. In terms of giving feedback, the province sends it to the district, the district sends it to the municipality. Through the municipality, the health facility that needs to be given feedback, the municipality gives feedback to that health facility. There are problems, there are mistakes, and the feedback comes to correct it. Likewise, giving feedback in the monthly meeting, semiannual review meeting and annual review meeting carries on.

I: Do you discuss by opening DHIS-2 in monthly meetings of the Municipality?

R: In our every monthly meeting we discuss about which institution has reported and which institution hasn’t reported by opening dashboard. Likewise, in the same meeting, we discuss about the data of which indicator of which program at what institution is not correct.

I: At the district do they come for monitoring in the health facility from the province level?

R: As far as I know, they haven't come for monitoring and supervision just for the DHIS-2. There hasn’t been any on-site coaching in DHIS-2.

I: In your opinion, what is the interest of administrative and elected representatives in DHIS-2 at the municipality level?

R: Don’t know about the condition in other municipalities but the chairperson of our Shahidlakhan rural municipality emphasizes and moves towards this kind of paperless program or digital program. He also mentions that we need to do it properly. He is moving by saying that we have to do it in our time and we have to do it.

I: So, they possess literacy of data?

R: They do have more data than we have. He also has the data that even we don't have.

I: What thing motivates you to use DHIS-2?

R: Now, after working in the organization, work must be done. Also, trying to deal and compete with technology and moving with the technology by utilizing it has motivated me. Even today, some friends see a computer and think of it as a tiger or bear. No matter how much training is given to them, they don't know it. Even if they are told to do it, they don’t agree. Even now this is happening in many organizations. Those who try to deal with and use the technology, they will move forward.

I: In your experience, in the coming days what and where is the necessity of the training in DHIS-2?

R: According to the way in which the program moves forward, there should be a training arrangement and it should be provided to those who are interested in taking DHIS2 training. If possible, if there is more than one trained person in the health institution, it will be easier to run. It is easy when you can ask for help from your friend when you are busy.

I: Are DHIS-2 related manuals or any other learning materials available in our institution?

R: As much as is inside DHIS2 will be taken out and utilized

I: Where and how has the data from DHIS-2 been utilized?

The data from DHIS-2 gets reviewed in the municipality then, in the district and the province afterward. The other thing is that it has made it very easy in our annual and quarterly review meetings,

I: How much have you been utilizing data in the decision-making process, planning and in programs?

R: Almost every program of ours comes after planning from the health section of the Municipality. Sometimes conducting programs at the field level e.g., deworming program, Vitamin A program and immunization program, the weight of the child is measured, immunized and target vs achievement of people receiving services annually is seen and if the indicators do not have target VS achievement, activities are carried out accordingly.

I: In your experiences, what suggestions do you give to make DHIS-2 more effective in Gandaki Province?

R: Till now everything is good. It would be better if we were able to use DHIS-2 in every district and every health facility of Gandaki Province uniformly.

I: Complaints regarding overreporting and underreporting comes from time to time. Do you have any experience regarding this?

R: It is like this, if we do two, we get two, if we do four, we get four. Overreporting refers to in what way we report and how much time we take reporting. We need to complete this work and if we click on complete overreporting and underreporting happens. By cross-verifying what matches with what and entering data by understanding and thinking then overreporting and underreporting does not exist. DHIS-2 is a machine, it works as per our operation. Overreporting and underreporting is our skill and depends upon ourselves.

I: Have you been practicing developing progress charts and displaying data in your health institution?

R: Yes

I: At last, do you like to add something about DHIS-2 that I might have failed to include?

R: The DHIS-2 program is very good. It will be good if DHIS-2 could be launched in every place and the health institutions start reporting online.

We have got a target population of 250-300 but even 90-100 people are also not found in the field. We have got high target population but the actual population is low. So, if online reporting happens if not in 1 year but at least in 5 years the data will match. If there has been misreporting, we need to think that there is a place for data verification.

I: Are eTB, eLMIS and DHIS-2 good when separate or are the respective three better when combined?

R: Making separate is easy. If combined in one, it will be unmanaged.

1. **IDI with DHIS2FP_ Taranagar Health Post**

Gorkha Municipality

Sr. AHW

Female

I: Form when you are using DHIS2?

R: I have been using it since the start of this fiscal year.

I: Which data you are entering in DHIS2 currently?

R: we have a monthly reporting form which is 9.3 and we are entering all the data that have been included in 9.3. We enter all the program data that we have conducted.

I: What is the situation of recording and reporting in your organization?

R: umm……….. recording and reporting is on time and complete

I: Which one is easy paper based recording and reporting or DHIS-3 based recording and reporting?

R: Paper based recording and reporting is traditional way so DHIS-2 based recording and reporting is good according to the current technological era. sometimes it doesn't work it may be its large size or other problems. sometimes we are busy we should complete the entry in time but it doesn't work. we have to enter data 5 to the 7th of every month but in these reporting days most of the time server goes down, not open when required. besides these everything is good in DHIS2.

I: Are there other problems with using DHIS2?

R: There are no particular issues at hand, as we have been entering the necessary information without difficulty. In fact, I've found it to be quite effortless, especially when compared to the previous process of manually tallying data by flipping through monthly pages for semiannual and annual review meetings. However, with DHIS2, we can effortlessly extract and analyze data according to our requirements.

I: do you use Pivot table?

R: yes, I use it quite a bit.

I: Are you physically visit Municipality for monthly reporting or send a copy of monthly report in paper?

R: We do the entry work from here, but the monthly report is run every 3rd of each month in the municipality health section and we also attend the monthly meeting.

In the past, it was not necessary to submit a copy of the monthly report to the health section, but later they requested a copy of the monthly report for their own use and reference, now we submit a copy of monthly report to health section.

I: have you faced any other challenges while doing entry besides server problem?

R: Specifically in data entry, there are no any other challenges.

I: Have you found any differences in data quality after using DHIS2?

R: DHIS-2 shows the exact data, there is less chance of mistakes while entering data. It shows the last month's data so there is no need to think about the last month.

I: is there any problem in internet and electricity?

R: No there is no any problem of internet and electricity.

I: If there are technical issues or other problems in DHIS2, where and whom do you report?

R: Firstly, we discuss it with our friends, then if the problem is not solved, we call the health section and they help us to solve the problem.

I: Do you receive feedback from the health section, district health office, and province?

R: We have received support and feedback. There are no big problems. if the problem occurs, we will ask for solutions.

I: Some say there is a little mismatch between recording and reporting forms in paper and DHIS2, is it right?

R: We do enter all the data. Some forms are extra in the DHIS2 in comparison to paper which we do not fill and leave unfilled. Some forms are already locked, which we don’t use.

I have been using it easily, It may be due to I haven’t operated it in depth.

I: What is the situation of monitoring and supervision from higher level?

R: um…… They did not come here specifically to observe DHIS2. Nevertheless, they participate in various health programs at the Palika and province levels and discuss and monitor data. The health section offer feedback and suggestions to us, pointing out missing data and questioning the absence of certain information.

I: Who operates DHIS2 when you are on leave?

R: ……smiling….. Until now, I have not required to take a leave when it's time to report. However, if there ever arises a situation where I need to be absent, there is another staff member at the office who is trained in DHIS2.

I: when did you receive training?

R: I received training in Shrawan month of last year.

I: Have you received training manual and learning materials related to DHIS2?

R: I haven’t received training materials. They said training manual send to office but not received yet.

I: What are the necessary trainings in coming days?

R: We have gained some knowledge and are actively engaged in our work. If we receive additional refresher training in the near future, it will greatly enhance our proficiency in operating DHIS2.

I: In your experience, where and how DHIS2 data has been utilized?

R: We discuss about the target vs achievement of different program using DHIS2. In case the programs have failed to meet their intended goals, we also analyze the factors contributing to this outcome. For instance, we examine the attainment of targets related to the number of users in family planning, antenatal care (ANC), and postnatal care (PNC). Subsequently, we devise programmatic strategies to effectively reach these targets.

I: In your experience, is there any program that Palika has developed based on evidence?

R: There is a program focused on nutrition and growth monitoring that involves setting up a nutrition corner at health facilities. Previously, a health post achieved good results in growth monitoring and as a result, they were granted a nutrition corner. This year, we have also excelled in growth monitoring, and as a result, there are plans to open a nutrition corner at our health post.

I: What motivates you to operate DHIS2?

R: ……………. Using it is enjoyable, and it is simple for data entry with compared to Talley. Additionally, analyzing data is effortless and can be performed whenever necessary.

I: suggestions for improvement?

R: Perhaps I am at an early stage or lacking a comprehensive understanding. I am unable to offer any suggestions.

I: How interested are the local representatives, and administrative staff in DHIS-2 and evidence-based decision-making?

R: They are positive. Our ward president is knowledgeable about the health sector and health programs, and he actively takes steps to initiate improvements in health.

We frequently organize meetings and invites them as well. They participate in the meetings and inquire about the status of the health data.

I: Is there a practice of presenting health data in health post?

R: Yes, ……. pointing to the wall……. There is a flex of immunization data. Similarly, we have safe motherhood data chart in ANC room.

I: Thank you for your participation

R: Thank you

1. **IDI_KahuHP_DHIS2FP_PMC_Kaski**

Pokhara Metropolitan

Public Health Inspector

Male

I: When did you start using DHIS 2?

R: It’s been a long time …since 2075 or 2076.

I: What types of data do you enter in DHIS 2?

R: In DHIS 2 we enter the data of 9.3 indicators.

I: Beside that, Covid or Lab …any?

R: Now we have started looking over covid too ...Laboratory is not available here. And then at DHIS 2 we enter the data of 9.3 including covid vaccine at different phase. Additionally, there are different FCHV App ...We have entered that one too.

I: And what about the situation of recording and reporting?

R: Its very good …it is 100% and even timely. Here it is 100% reporting.

I: How do you feel about using DHIS 2? any experiences?

R: It feels so easy, our work has been easier due to DHIS 2 ...We don’t have to use our hand anymore ...and has been easier to find the recording or reporting. Because of online system it has been too easier for reporting. It is also easy for revision of report, if there is any mistake over here, and received any feedbacks.

I: From where do you receive feedback?

R: From health section of Metropolitan. There is monthly review too, Feedback from DHIS2 is still not very influential. It hasn't had much of an impact because there isn't a competent employee in the section to oversee it. But we discuss on indicator during monthly review and if we find any errors then we edit it later on and submit it.

I: Have you had any problems during data entry?

R: There are no such issues. Since we are doing it on time. When someone enters, they must be validated, and during that validation, there is one indicator of safe motherhood where the indicators of iron supplements and 4 ANC are out of balance. It asks for the balance, and an amendment is currently being made. Online, it is requested that both sides be balanced, but to our knowledge, this is not required. Beside this, we don’t encounter any other issues.

I: Okay so what about problem related to server?

B: Sometimes it happens, it is quite slow may be because everyone uses it at once. Because I had to enter the data timely, I had to travel another location a few times. I also completed this task in the evening. so, this is the condition.

I: Any difficulties during data entry?

R: There is no such issues.

I: What about you? Have you faced such difficulties?

R: No, we haven’t faced such difficulties.

I: During Analysis?

R: In analysis definitely there. When interpreting, there is. We are giving it a try. We have completed the interpreting trainings. I first attended the training, followed by one of the other female employees. Other staffs are also operating it but I'm unsure as to whether they use it perfectly or not.………… So that’s the problem ...So there is no interpretation ...there are lots of app. There are pivot tables and data entry on the app, but we aren't properly using it. on reality, the app is merely there to show off.

I: Have you completed all of the related trainings?

R: Not all, there were a lot of sessions and discussions on data entry and pivot table. However, not much on interpretation. We have requested on refresher since all health workers have to be included in DHIS2 trainings, everyone should know about it. Health workers should know what is there in data entry, interpretation and reporting of DHIS2.

I: So, do you get any help to use DHIS 2 from section, district or province?

R: No there is no such help to use it. I took the trainings first then last time one of my colleagues was trained. Though, we only use the data entry. It has almost been 4 years of training still I have not received the refresher training. So, whatever I know I do if I am unable to use, I take the help from YouTube .so I am learning in this way.

I: Sir, are there no any manual, is there anything like that?

R: No manual. For DHIS 2, the manual has yet to come. During our trainings, there was only one sample, however, there was no manual.

I: I have heard that it can be downloaded from the site?

R: I just got to know about manual from you.

I: I heard about it from two places

R: Just got to know about the manual.

I: Sometimes we enter only few data but it shows more than that in there ...is there any such conditions?

R: we have not faced such situation. It gives same amount of the same data that we enter. But one time I faced the similar situation during reporting, During the immunization review I did the reporting manually by using the formula. But it didn’t match because of which we present the wrong data. And after the presentation we look over the situation and found that there was a wrong interpretation in DHIS2. There was problem in formula to calculate vaccine wastage rate, dropout rate.

I: It is said there are difference in the HMIS 9.3 form and DHIS 2 form ...is it?

R: Yes recently, So I do only the reporting of the things that is in the DHIS2 form. There are more than there are, in that form….so we only report the things that are available in this form. There is double entry in this form in case of morbidity. It ask to enter about the symptoms ...so I print this one. I have brought the paper form but have not done the entry …have not fill that form yet.

I: They say, it is not based on new form and so entries are missing?

R: During the monthly review meeting, we discuss on this matter, so we conclude to make this form available in DHIS2 authorize rather than paper so we didn’t use paper form …so we print this one.

I: Is there any changes in the quality of data after the use of DHIS 2?

R: Yes definitely, yesterday there was RDQA, and it was found that our data was 100% correct. There was no variation and it was 100% same. There may be various reason being RDQA low. But data quality was 100%.

I: who did it?

R: there was no variation. Health section ...visited and did it.

I: And then it is easier to oversee the data, performance, right?

R: Yes.

I: Is there any changes in the health service delivery due to DHIS2?

R: umm…in services …There is low utility I case of services ...We enter the things that we do. Because of interpretation being weak there is no use in services delivery. It should be utilized in service delivery by interpreting data. Since high number population is seen in lower land and few number in upper part of the area. There are different big hospitals are there in this ward, so it is hard to interpret data and use it for service delivery.

I: So, you may plan the activities so at that time do you consider these data?

R: Yes, we do

I: Any example ...?

R: We take the reference. During presentation we found that, indicators of post ward, immunization of our institutions are very low in comparison to others. So, we use data to plan awareness activities and other events are also planned based on these data.

I: Is there any system of performance evaluation from the section office?

R: No there is no such things till date.

I: What do administration and ward representatives think about the use of data literacy?

R: No!! They have never asked about it yet. They haven’t asked.

I: In order to plan or related activities …?

R: No. They have not asked about the data. We give them our planning and they use it. Maybe they have faith upon us so. They themselves haven’t ask about the data.

I: Do you have any medium to share your data?

R: After the arrival of new group there are no such things, we present in the yearly review and provide them a print of a hardcopy. After the arrival of new representatives, we haven’t given to them.

I: What motivates you to use DHIS 2?

R: Since this is online system and it is easy to edit. We don’t have to use paper any more, no need to use Tipex to correct the data. Data are easily available at any time in a single click. We can edit it from home. We can see it in different format like pie chart, bar diagram. It is paperless and so is easy.

I: Able to see the data of previous year too, right? If there is any error or mistake, do you receive the mail from province or Palika?

R: Such things have not happened yet over here. I see the messages in the message blog. I can see the text for other organization regarding it. But have not seen the text for our institutions. I could find that other are having errors in their reporting through the text.

I: So, they say about the mistake through message or email?

R: Through email too. One time we had received the call asking for the verification as there was some difference in the number, but email ...I don’t remember ….

I: Any necessary training besides refresher in DHIS 2?

R: Trainings are required to operate all the application available in DHIS2. Every health worker should be able to use it equally, interpret and use it. Although not all the applications are mentioned here, it is essential to employ them all.

I: So, what do you think what should health section should do in order to make DHIS 2 more better or more influential?

R: We have electricity problem over here, if there is power cut off, we are not able to complete it ...so there is problem in back up ...and even there is problem in internet. If there is storm I, then we immediately have a problem of internet. It doesn’t work properly. It creates a lot of disturbance. Internet is not so fast ..but works ..if power is cut off then we had a backup problem .So it the problem.

I: So, to make DHIS 2 more better what should Gandaki Province do? Any suggestion would you like to give?

R: Instead of Province, municipality health section should do, ……

They should regularly monitor the activities. There is no manpower for this in the section. There is data assistant but I have no idea whether he has knowledge on DHIS2 or not. Section should have the provision of feedback system by having comparative analysis between the institutions. They should give the feedback if there is any weakness. Some may be having problems while using it, so such manpower should be there who could look over these things. Since the employee over there is weak then the periphery so it is difficult.

I: So that means they don’t discuss about the data from DHIS2 during monthly meetings?

R: No, they don’t open the DHIS 2. They carry our hand book, as we provide them the hardcopy and we discuss from that, they have never visited to DHIS 2 site while discussion

I: Only after discussion you enter the data?

R: No, first of all we enter then only we discuss at there. If there is anything that needed correction then we edit those data next day. As we can edit the data within 15 days.

I: Okay Sir, these were the things I wanted to know for my study regarding DHIS 2.

R: We do on time.

I: It is easier right …

R: Yes, it is quite easier than hand.

I: you use ETB?

R: It is not available here.

I: ETB, eLMIS?

R: There is eLMIS.

I: What do you think, whether ETB and eLMIS should be integrated together or they should be separated as it is?

R: Sir, if it is possible then it’s very better than working separately. It is better if it is integrated. Maybe It will be integrated in near future, to this date it is separated.

I: Province has asked me to inquire about it.

R: Okay... It is better in integrated way ...or through the same medium.

1. **IDI_LahachowkHP_DHIS2FP_Machhapuchre_Kaski**

Machhapuchre Rural Municipality

Sr. ANM

Female

I: Mam, when did you start using DHIS 2?

R: Its about 2 -3 years ago. – Use of DHIS2

I: And what sort of data do you enter in DHIS 2?

R: Sir, we enter monthly report at DHIS 2

I: Okay

R: Monthly, programs like safe motherhood program, child health program – data entered in DHIS2.

I: okay

R: Similarly, our daily patients report. right ...in fact we enter everything that DHIS 2 ask for.

I: What about Covid ...?

R: sometimes we do enter the data of covid when covid vaccination is going on.

I: What are the situations of DHIS 2 reporting over here?

R: Sir you know what happens …... (background noise) … We enter the data into DHIS 2 and sometimes errors occur, these errors are also reported with reporting but we cannot correct them because the data is locked after reporting. – system error

The mistakes are noticed later, once the data have been reported, it is quite difficult to repair those errors.

Such issues do arise; occasionally, servers are unavailable, which prevent us from timely data entry when it is needed. These issues are now present. – Issue with server

I: Other than the server, are there any other issues?

R: Only this is the issue at the moment.

I: Is there any problem during data entry?

R: There are currently no issues with the entry process.

I: Any problems or difficulties during validation?

R: No, it has not happened yet, but sometimes it does create difficulties ...sometimes when we do run validation and complete after data entry in the sheet, the system doesn’t show the entered data later at there. In the previous month, I experienced a similar issue.

And so, I got a feeling of having a double entry …but later on it doesn’t show the double entry.

I: were there such things of having DHIS2 shows more data than the data in the paper?

R: Sir, by mistake such things may happen ... but I have not found those mistakes here, in fact there are no such entry

I: Most of compliant that, we enter relatively few data, but it displays more data?

R: No, I haven’t found such things.

I: So, such things have not occurred!!! And do you analyze the data?

R: Sir, At the review, we do the analysis

During the time of review, we take the data from there. If we take the data from monthly by tally, it creates the difficulties as we need to see every papers/ detail. And with only one click we can get those data from DHIS 2 ... (Laughter during the conversation) …... So, it makes us easy.

I: Anyway, you are using it right ??...And what about the Pivot table?

R: Yes, sometimes we are in need of it. …yes, we take the data from the pivot table.

I: So. .do you find the data yourself??

R: Yes, we do analysis from data set report, Pivot table. We use both of them

I: Are there sufficient materials available at the health post for the use of these tables?

R: Yes sir, there is one computer. If they provide us additional number of laptop or computers it would have been easier.

And then if trainings are provided to all other staffs, then it could have been better and easier right. If there is only one and then, when one employee is transferred or trained staffs on leave or absent then it is difficult for us to enter the data on time …As data are said to be enter timely. So, if he/she went to meetings or trainings then time is delayed for the entry and after certain time DHIS 2 is locked and it creates the problems ...And it is difficult to manage online or offline.

I: And are there internet or electricity issues?

R: No, to the date it’s okay sir. If solar is provided for the back up then it would have been better ...otherwise it is okay sir …sometimes it disturbs.

I: Yes, backup is also important. How often to you get support from municipality, province and the district

R: for easiness?

I: Yes, for DHIS 2

R: Yes, they do ... sometimes they come and teach us …when we are doing alone it is difficult for us sometimes, data sets are locked, sometimes mistakes do occur, so sir asked us to call if needed help.

I: Where do you ask?

R: Health office or health directorate.

I: here at municipality?

R: They are there at the municipality …. sir…ump. We ask at municipality too ...in fact we are closer now in the same building …in the next door. Because of that it has been easier for me …first we need to ask to our own authorities …if they couldn’t help us or if they don’t have idea then we go and ask to district and province. We don’t directly ask them first.

I: So, you get the feedback calls from higher authorities?

R: Yes, sometimes we do receive the call saying this one is not okay. Sir, even We do eTB from there. And also, there is eLMIS. It had been easier than before after its use

I: so, I will ask about it … Is it okay to have eTB and eLMIS in different form or it could have been easier if they have integrated them together?

R: Definitely it could have been easier, …. it’s okay to have them as a program base too ….

I: So, you are not having difficulties?

R: No ...not so difficult ...itis fine. Here we don’t receive lots of cases ........ So, it is okay.

I: How much easier has your work been since using DHIS 2?

R: We need to do a lot of paper work right…and next one … (background sound) …we have daily reporting. We have daily patients report, daily program report. So, I think, if there are such software where we can directly report them at DHIS 2 then it would have been easier.

I: Because of DHIS 2 it has been easier for reporting and to generate data, is it?

R: Yes, it has been, because of the software we can easily get the data whenever required, we don’t need to come office to get them ...if anyone ask about the data, we can generate it from the computer. It is easier.

I: do you present the data in the monthly meeting?

R: At monthly meeting we carry the hardcopy. And if the authorities have to discuss then they look over those data from DHIS 2 themselves.

I: Does the municipality have a system of looking over those data …?.at monthly meeting…?

R: yes, there is. In front of the authorities like the health coordinator, administrative officer, we present our statistics. If they are unavailable due to their hectic schedule, we look at the hardcopies of all other institutions. And during the review we discuss and look data from DHIS2.

I: When it comes to the data, how interested are the representatives?

R: If they are expert in data, they speak about it. If they are from administration and have previously work on data then they do speak about it otherwise they don’t.

I: Are future plans made based on the information now available from these data or on the agendas? What situation exists in this regard?

R: If asked also… they don’t leave their own matter ...but also … (laughing) …I don’t want to say much on this.

I: At least you need to present your data…. do you aware them regarding the situations in your presentation?

R: It is said during the presentation …

I: Then…

R: We aware them regarding the situation …the place where we are in, the conditions …. we do comparative analysis too….

I: Have you find any changes in the quality of data after the use of DHIS 2? Or is it same as it was before?

R: It won’t be same as it was before right sir? we enter the data …we still do use the hardcopy …so we enter the data …and then we could be able to see the data after registration only …right??

I: Oh yes… So, the perspective of the use of data in decision-making has evolved, right...??

(Few sec silences during conversation)

and is there any trend chart or monitoring chart in your organization?

R: monitoring chart …?

I: Yes

R: Yes, there is

A: Do you tally it or based on DHIS 2?? Or how do you do??

I: We do it from register .1^st^ we register at 9.2 then 9.3 or both. We enter data at there ...since we are not around the computer all the time …chart is not there all the time …

I: What motivates you to use DHIS 2 and eLMIS?

R: It is unnecessary to hurt hand...no need to go through paper...and the electronic equipment brought here by technological growth is this computer. So, it has been simpler.

I: As there are some differences in between the DHIS 2 form and the manual form ...so how do you enter the data then?

B: Yes, it is, and we start tally right away. We experienced issues with Sharawn because the format and code were altered in that month. Because of this, Sharawn's reporting was terrible and full of errors. We didn't know what the DHIS 2 request was. And in our 9.3 there are specific things, like the requirement to fill out a form for the 60 to 69 age range on the front paper/form.in our format, however DHIS 2 also asks for data older than 70 years. Therefore, these issues are not addressed. and ICD codes are also lacking. DHIS 2 requests information about items that are not available in paper format... And we have to keep looking for things that aren't in hardcopy.

I: So that means you manage to enter the missing data… at least somewhere?

R: We didn't forget to enter the data, sir; at least, I don't think we did…… (laughter)…... It could also be miss. I'm not sure.

I: Is there any form which are not necessary but needs a complete validation. necessary to complete ...?

R: Yes

I: Is it necessary?

R: It might not be here but in other places there might be ...so that may be the reason of which they are included in the program. And so, I think they can’t be removed ...right...

I: There are thing that is not necessary at the health post ...or the services out of the health post …. that’s needed to be complete… is it?

R: There are lab services at PHC rather than here. PHC should therefore do reporting. And they are not accessible here, and I recently learned that this too required full validation.

I: According to your experiences, what suggestion would you like to give to make DHIS 2 more better in Gandaki Province?

R: Everyone should be provided with DHIS 2 training ... they should be provided with computer or laptops. Sir, there should be similarities in between the DHIS 2 and manual format.

I: When did you attend the trainings?

R: I took DHIS 2 training about 2 to 3 years ago but I started using DHIS 2 later on.

I: So that means you started using DHIS without training?

R: No, I only began using it after the training. You know what happen, when our health in charge was moved, I decided to attend the training, but DHIS 2 was not made mandatory there. Later, other incharges started using it, and I was then moved to Lahachowk. Sir didn't use it at the health post, so I must use it. I began utilizing it and picked some tips from others. Now I enter the data myself.

I: Do you have any materials or manual for using DHIS 2?

R: At this moment, No!! It is complete based on training.

I: So, refresher training is required ...besides refreshers, is there any training you want to seek?

R: I have no idea sir...!!

I: There are lot of function inside the DHIS, like Pivot Table, report and so on …do you want any such trainings?

R: These will come under the refresher training. Once we star using it or start entering the data ...we will get to know about such things

I: To some extent, it has been easier to provide health service ... is it?

R: Yes, it has been!!

I: Is there a system of using data to look over the performance in the municipality…. (Sound disturbance) …in the health post …is there such things?

R: Our health section looks over it.

I: Does it monitor the activities and the performances?

R: Yes, it is!!

I: Okay!! Thank You so much. If there is anything left to discuss on regarding DHIS 2, is there anything you want to share based on your experience’s then could you please share it?

R: Ummu... it’s okay sir …I don’t want to speak furthermore

I: Okay!! Thank You

1. **IDI Health Post-Hupsikot-DHIS-2**

Hupsikot Rural Municipality

Male

Senior AHW

I: How long have you been using DHIS2?

R: I have been using DHIS-2 since last 3 years.

I: Now what types of data you are entering in DHIS2?

R: All data from 9.3 form and data asked by DHIS-2 are entered. COVID related data are not entered regularly. Nearby we have PHCC so data are entered from there.

I: Is recording and reporting done through DHIS-2?

R: Yes, it is done through DHIS-2, one hard copy of 9.3 is given to Palika. And two photocopies are kept here. Plus, I am entering data in DHIS-2 from office. If we were not able to enter data then, other will do from Palika level.

We mostly enter data by ourselves. We have monthly meetings by 5^th^ of every month. Data will be arranged by 5 and it will be entered by 9^th^ of months. If it gets delay then we finish data entry by 5,6^th^ of months.

I: How easy DHIS-2 had made in recording and reporting?

R: DHIS-2 had made very easy in reporting. In earlier time we had to do from manual basis, where conducting annual programs or other programs, all necessary data management was bit difficult. But now, any of the program data like immunization program, can be found easily in annual or semi-annual basis. For participation in each and every program, if we need any of the data we can easily find, print out and verify data from anywhere.

Now it makes easy in every aspects.

I: Does it makes easy in monthly, quarterly, or other review meetings?

P: Yes I had told it earlier. We do monthly, quarterly, semi-annual or annual reviews by operating data. If any program should be done separately like if we want do program related to nutrition then whole annual data of nutrition can be operated easily.

I: Does it had made easy in evaluation of program trends?

P: Yes, it is easy for us to look at the data of the program which had already been entered. While doing the work there is always a chance to commit error. But we don’t have authority to enter zero if the data is blank. Server gets hang when there is overload. This is a problem of whole nation not only of us.

Sometimes, while entering the data if we found zero, there is no any need to fill up the data so we get confused.

As per my experience, till this date when I tries to look back at the data entered 1 or 2 months ago, I remember I did it, but while checking it back, the data was not there. This has happened many times, not once.

I: Do you experience same problem now?

P: yes, we face this issue today as well. While entering the data it is right there, at that moment but after checking back, it has been lost where we entered before. But I think this is the problem of system. While taking out the print, it shows system error.

I: Is it possible to see coverage, Target v/s achievement?

P: Yes, we can look back up to 2 to 3 months data. Like while viewing data of Asad and Shrawn if some space is left to filled up then, it gets locked. We cannot do anything if it is locked. Later, by informing to the higher authority the lock is opened. Yes, this happens often.

I: Do you take decision or conduct program as per the result of the data?

P: Yes, we do. During Annual and semi-annual review program specific program such as Immunization program, Safe-motherhood program, and Nutrition related program are conducted as per the need. Data can be taken differently as per the situation.

Data of last 3 years is reviewed in Annual review program, and for semi-annual program data of last 3 years is taken, suppose of BCG and achievement on it is reviewed and Comparison is done if target has been met or not.

I: Did you get any difference in data quality?

P: It is problematic when there is an error in the data, rest of the thing in the system is fine. It is bothersome when the data of month back is blank.

I: When is the data is being utilized in Health Institution?

P: It is used to find out the data of the specific program. Every program data has been utilized. By looking at the nature of the data the program is conducted.

I: Is data sharing is being Practiced?

P: yes, Immunization data is been shared and another program as well. Also, we present data by making it in the chart.

I: Do you make Performance evaluation of your Institution?

P: Yes, we do.

I: And, how is it helping in delivering the Health services?

P: There are many programs coming forward. There is also a provision of providing Quality services. DHIS-2 data cleaning Quality is also increasing. Data should not be lost in DHIS-2, rest of the things like reviewing the previous data is been quite easy. We should not search the file manually in the cupboard to get one in next month, whatever we need we can get easily from DHIS-2.

I: What things motivates you to run DHIS-2?

P: They ask us to enter data in DHIS-2 previously, and then we started doing it. After using it, it has been quite easy for us to view the data of any time. Also, the data can be viewed by the higher authority by sitting in any other place as that of Kathmandu, Biratnagar. Moreover, data can be checked from MOHP as well if needed which may also helps in improving quality of data.

I: what are the challenges in DHIS-2 which you are facing currently?

P: As I already mentioned, there is a problem of system error, data get lost on its own due to system error, So, people from higher authority must take a look at it working at this field. It is also followed up in the district. If some error is seen in the data, we asked them to take a look at it and then they sent us that the information that the data entry is error. We sometime correct it; this sort of system error occurs often.

Another problem is most of the Health facilities enter the data 7-8 day or 9-10 day of the month. Due to this, the server does not run, DHIS-2 out of reach information comes out.

Whenever I get free, we enter the data, as we are not the person working in this field. I am a health worker and I also have to provide health service to the patient. So, when we try to open DHIS-2 the server could not load. But sometimes it gets open, and then we enter the data. We do this at home as well.

We can see many problems regarding system.

I: How is the condition of internet and electricity?

P: There is not much an issue of internet here. We can manage, like if internet gets disrupted here due to heavy wind, I often take laptop at home and work on it. Sometimes, we can work in municipality as well as the speed of internet is good there.

I: Do you get any problem in data validation and data analysis?

P: There is no any issue in infrastructure, as we have Laptop, Desktop. Sometimes, the problem is in server rest of it, we can manage.

I: What kind of help do you get from Province, Municipality?

P: Whenever we get confusion on using the tool there is a provision of calling them directly. Also the authority from the district and municipality, check the data of whole district, if they see any kind of problem they tends to inform us and ask to make correction, we make it.

I: What are the Monitoring and Supervision status on DHIS-2 from higher authority?

P: They do not come for Monitoring and Supervision directly; they do this through online. If any problem is being seen direct call does happen.

I: What is the status of Training? When did you take training?

P: I had knowledge of entering data in the DHIS-2 before training. Also, I was having a wish to take training on it so I took a training of 3 days on it.

I: While attending the training did you get any training manual or any other learning material?

P: They didn’t provide us any book, but they provide us with a print copy which contains important information about the training. And rest of the things we look at the diary while receiving the training. Now I can say that, practice actually helps to make us perfect on it. The more we use it more we can learn from it. I once left to use it for 3-4 months I got confuse. But I am fine now.

I: What kind of training are needed in upcoming Days?

P: Regarding DHIS-2 refresher training should be provided time to time. In 6 months, 1 year, 2 years whenever possible, training must be given. I once have taken Basic Training. But now the system process changes frequently. There is a change in before and now 9.3. it has to be updated. As per HMIS 9.3 in DHIS-2 should also be changed .so that there will not be any problem in data entry.

I: So, the form in Paper and in DHIS-2 form do not match with each other?

P: Yes, there is a difference. Similarity between the form must be ensured so that while entering the data it can be easier. If the context in paper form and DHIS-2 form is different or do not match up it then gets difficult to enter the data in the tool. Also unnecessary data should not be mentioned there in form. At health post level required data is different then that the data of Hospital data. So that as per the requirement data should be entered differently in Hospital and Health post differently. The form should be open accordingly, and in health post setting basic things should not be ignored and included.

I: In Municipality level how the data is being used?

P: Yes, data is used in Municipality level as well. Presentation is done by using the available data. We take laptop there at municipality and by using internet we take out the necessary data and present it forward to all. It is not so necessary to read out now, we can present by using technology in this era. It is not possible to do data analysis now, we enter the formula and present the data directly. So, this way data is being used in Municipality level as well.

I:

P: We face technical issues while the server is down. Also, the blank space after entering the data can be seen. So, cross checking on this issue is quite necessary. Eg; Is it possible to do cross check the data of 1-2 months back?

It is mandatory to cross check the data of 1-2 months back. We enter the data and press ok and complete it but the same data is lost in between after checking back. so, this issue must be solved. It should not be locked rather it should allow us to make correction if needed.

Eg; During the time of annual review, I got to know the error of months 2-3 back, let’s say of the month shrawn. I see one data entered is shown error, whenever I tries to make it correct it DHIS-2 pops up “data issue” and do not allow to correct it. I know that it needs to be make a correction but it does not allow. So, this can be the issue sometime. Like that of in 9.3 report can be taken out and after making the correction we can present it. It is difficult when the system is locked.

So, once the DHIS-2 is opened through one ID, data needed for Annual review, semi-annual review can be taken out and viewed but when it gets locked it become difficult to extract the data. So, this is the simple problem, and can be solved as per time.

I: Thank you! Sir

P: Ok! Thank you.

1. **IDI with DHIS2FP_Rajhar Health Post**

**Devchuli Municipality**

**Male**

**Sr. AHW**

I: Namaste. I am doing research on DHIS-2 in five districts in Gandaki province. In terai, I will be doing in Devchuli and in one gaupalika. I would like you to share some experience while using DHIS-2.

R: DHIS-2 was used by municipality previously. Later when health-related program initiated, after taking training for 3 days, we started using DHIS-2. In DHIS-2, we do data entry only so, there are not much problems. But we face problems during reporting while making pivot table. We are not fully capable in using DHIS-2. We are in learning process. We have been doing data entry in DHIS-2 for about 2 years.

I: What type of data are entered in DHIS-2?

R: In DHIS-2, we enter data of HMIS 9.3. Also, we enter data of TB records through TB register.

I: What about Covid records?

R: We do not keep records of Covid here, it is done by municipality.

I: After using DHIS-2, is there any change in recording process?

R: Yes, there have been many changes. We compare data while recording and reporting. DHIS-2 shows all the weaknesses in terms of trimester or annually. We keep records and make conclusions. Specially, while watching DHIS-2 in laptop or computer, we can easily compile DHIS-2 and TB register.

I: We are doing paper-based reporting as well?

R: We send a copy to municipality (paper based) and original copy is kept here which is entered in DHIS-2.

I: Is it ok to perform both of these works or you wish to use only DHIS-2?

R: For recording, government’s 9.3 reporting is essential. A copy has to be filled up. And the other copy has to be submitted to municipality. Previously, three copies had to be made for municipality, district and health institution. But now due to DHIS-2, we do not have to submit to district. Original copy is kept in health post and the other copy is reported to municipality. It has become easier for us as there is no need to carry the original documents. We have passwords and we can get access to data wherever and whenever required if we have internet. This service is nationwide.

I: It is easy to work on DHIS-2 then?

R: Yes, it is easy to work on DHIS-2. We do not know perfectly to work on DHIS-2 like making charts, diagram but we know how to retrieve or search data.

I: What about monthly and trimester meetings?

R: Yes, we go to meetings and submit reports. We enter and compile data in DHIS-2, search for corrections and improve it.

I: That means while going to monthly and trimester meetings, the data are retrieved and compared?

R: Yes, we retrieve those data. Even now if someone asks to find data of particular month or day which has been entered on the website, I can show them automatically.

I: You have told that it is easy to work on DHIS-2, then how do you make decisions?

R: We focus on targets and achievements. We find our weaknesses and try to improve it. This can’t be done by a single person, joint effort is required.

I: ……..

R: Though we use these registers for reporting, we give priority to DHIS-2 reporting.

I: How is the quality of data in DHIS-2?

R: We have used for quality in DHIS-2. There are no ICD board and have been repeated in some. We have to make corrections and make follow up for this as well. Many corrections are to be made. For our level, I feel essential things might be missing and non-essential things are many. For higher level, they might need for same level of data. These things might be lacking in DHIS-2. The format records that are not in 9.3 are here in DHIS-2 and those format records that are in DHIS-2 are not in 9.3. This has become uneasy. For example: Previously, in main register, 60-70 is not here but it is present in DHIS-2. There are differences in ICD board in 9.3. So, we have some difficulty while recording. Two three formats are not present. The column “Others” is not present. The non-diagnosed cases have to be kept in “Others”. We have been requesting for this frequently.

I: The other cases are not reported then?

R: They are reported but are not valid. They are entered in DHIS-2 but in other column. Due to lack of column, there might be under reporting or over reporting.

I: Has DHIS-2 helped in service provision?

R: I don’t think there is any use for service provision. DHIS-2 has helped us in reporting. This can show the records of services provided by the health post or the number of cases from which we can estimate the medicines required. But, there is no any relation of DHIS-2 directly with the patients.

I: On the basis of records, we can focus on services….

R: On the basis of records, we can focus on cases and medicines but they do not directly interact with DHIS-2.

I: Health education program is also provided accordingly?

R: Yes, health education program can also be provided accordingly. It helps to focus on appropriate manner by focusing on the age group, sex, sickness.

I: It helps to make our task easier?

R: Yes, it does. One of the main advantages is we can save data properly and can retrieve it whenever and wherever required. Previously the paper-based data used to get lost or become useless due to water or dirt. DHIS-2 has made the recording system easier. Data is reported from health post to municipality, municipality to district, and district to central office. Time is also saved and the central office can make decisions accordingly. Communicable diseases and outbreaks can be investigated easily and quickly.

I: What motivates you to use DHIS-2?

R: Using DHIS-2 is a compulsion. In my tenure, reporting was all handwritten and we used to do presentation by pasting on walls. It’s been only 2-3 years I have been using DHIS-2. Nowadays, presentations are done by making slides. In this new generation, these technologies are being passed. So, it has become essential to use DHIS-2. I may be happy using the reporting formats previously but it is good to make changes according to time. And, we are up-to-date in data entry in DHIS-2 and perform data entry on time.

I: U mean that this digital era is the motivation for using DHIS-2?

R: Yes, that’s true.

I: What are the challenges while doing entry or retrieving data in DHIS-2?

I: I don’t think there are any till now. It is a simple process. Even ordinary people can use this easily. During report processing, one might have to go out of track to see the report. There are many features but we are not able to use most of them. We do not have enough knowledge. We are capable of performing our task well.

R: There are problems in internet and electricity…

R: Yes, there is problem. Subisu is providing free internet otherwise our previous Adsl phone and internet has not been running since 3/4 months. Due to lack of electricity, we are not being able to use digital thumb attendance. This is not the same, this is due to lack of road access.

I: Until when subisu will provide facility?

R: They will provide facility for up to 2 years now. They keep asking when we will pay for the internet. They say we have to pay after 2 years. That’s okay we’ll pay. It is not an individual’s responsibility; payment will be done from the Nepal government. They have provided good internet facility and we have been working properly. The Nepal Telecom net has not been working for 4/5 months due to road problem. They will surely help in providing internet facility very soon. Due to Subisu, we are not facing much problem while working.

I: Do you face problem due to internet server being down?

R: Yes, we face much problem while reporting in DHIS-2. Sometimes, the entered data might also not be saved.

I: It happens most of the time?

R: It happens sometimes only. Sometimes, we might also not enter all the data completely which is our weakness. The data might be missing then. We enter data again and we check this time to time.

I: Is this due to lack of training?

R: Yes, training is required. Before, we lacked knowledge and used DHIS-2. But if detail training would be provided, we would be clearer.

I: What type of training is required then?

R: Training not only to do data entry, but training to make pivot table, reporting in pie chart, bar-diagram, list top ten diseases could by very useful.

I: Is there any manual for using DHIS-2?

R: I don’t think we need any kind of manual. They didn’t provide any manual to us that time. They told us to bring laptop. That time, I was able to enter few alphabets only. I took training without having knowledge and now, I am using it. I think there will be improvement while using DHIS-2 in the coming generation.

I: If there are any problems using DHIS-2, how do you solve it?

R: If any problems arise, we try to solve it through communication with our friends. We take suggestions from municipality or district. We have one personnel from IT at municipality as well. We do according to their suggestions.

I: How is the help that you get from municipality?

R: Everyone is doing their own task. If we have problems, we ask to the municipality. They are ready to help. Unlike other fields, I don’t think there are issues in health field. There is good coordination and communication from lower level to higher level.

I: How is the help that you get from ward members or representatives for strengthening Information system based on statistical data?

R: It is according to statistical data. Work is done through networking. I think everyone is promoting this. Every planning and presentations are done based on statistical data. This is assigned based on educational level of members of municipality. Previously, we used to paste handwritten newsprints on wall but now, presentations are done with the help of slides. Presentations are done with the help of slides in training halls of municipality and the one who does not know how to prepare slides are backward in this.

I: Opportunities in using DHIS-2?

R: If this will be used daily, it will be useful. If work is done based on levels, it would be better. It would better if data entry would be done based on levels separately for PHC, HP, hospitals and hospitals. Otherwise, there might be confusion for data entry from hospital level to lower level. This is better than paper-based data and should be prioritized.

I: What other improvements are required?

R: I don’t think there are any other.

I: The records in form and DHIS does not match?

R: Yes, we have been telling about this many times. Not DHIS-2, but the forms published by Gandaki province needs to be improved.

I: Are the local levels using the statistical data in health planning?

R: Yes, they use. Some are using and some are not. Overall, budgeting is done based on these statistical data prioritizing poor, disadvantaged, diseased people.

I: Is there anything I have missed in DHIS-2?

R: This is what I have experienced while using DHIS-2. We have been using this and will be doing as well. Please share if you have any suggestions.

I: Thank you for your time

R: Thank you

1. **IDI_MarphaHP_DHIS2FP_Mustang**

**Gharpajhing Rural Municipality**

**AHW**

**Male**

I: Which is our health post?

R: Our health post is Marpha Health post.

I: How long have you been using DHIS-2?

R: I have been using it for one year.

I: Have you received training before using it? Or you are using on self?

R: Yes, I had received Training on HMIS; and oriented little on how to enter data in DHIS-2 but I had not received training on DHIS-2 specific.

I: which data do you enter on DHIS-2 now?

R: We enter data of Master register, OPD register, and those which are service related from Health post. We enter those data.

I: I wonder if you are entering COVID related data?

R: The COVID has a separate portal for entry which is IMU. We only enter if someone gets tested from our HF s/he is either negative or positive. If there is no COVID tested we donot enter.

I: What are the problems and challenges you have to face/tackle with while operating DHIS-2?

R: I have not been able to entry data of this month as well. Data are available, but sometimes server is down and somedays system will not be properly functioning. This time as well I am facing problem due to data set not opening. It is continuously saying that data set is locked.

I asked to higher authority, they also said it might be problem from the center. So, it creates problem as server will not operate continuously/ uninterrupted.

I: are there any problems faced besides those you already reported; in using DHIS-2?

R: As DHIS2 is online portal, we use it only online. The main problem is that we cannot use it properly at time due to server being down and busy.

I: Are there any other problems other than this?

R: Yeah, besides server and internet problem other have not been faced.

I: For analysis, could you extract Pivot tables from the software?

R: Yes, if required we can download and construct the pivot tables

I: Is there internet and electricity related problem?

R: Electricity problem is not here but sometimes the internet problem and server down only. Thus, these created problem sometimes but now they are fine here.

I: Are the physical facilities adequate for running software there?

R: Yes, it mostly requires uninterrupted and strong internet. Internet is well managed and better here in our HF.

I: How much support do you get from the palika, district and province levels for managing and addressing issues of errors within the software?

R: We do have monthly meeting in the palika and we have review, feedbacks and suggestions from the peers and supervisors.

I: Do you open DHIS-2 during such meeting to discuss on those issues?

R: We donot open DHIS_2 there. We talk about the services related reporting ie. 9.2 and 9.3. We also use it to open the reporting status, to perform the pivot tables, data visualization, GIS those which are useful for us.

I: So, how frequently do you receive feedback and suggestions from the district, province level?

R: Yes, they provide feedback and suggestions from the RM as well as sirs from the district. From the district level they inform/feedback to RM and from RM they relay and brief us the information/feedback.

I: How much easy do you feel while working through DHIS-2?

R: Yes sir, it has been so much easy now. Before we had to entry the reporting form 9.3 and again extract it for the charts, we needed to note down all the details. Now, we can easily enter and get the information of the period we require. It has been much easy.

I: So, it has also been easy to review own works and monitoring as well?

R: Yes, sir.

I: How has it supported in data quality improvement?

R: I think the quality is similar as previous. Might be they have been upgrading the system. So, the server is not functioning well since last month. I have been trying now as well but it says data set locked. I had followed up. And they said we would not accept for 20^th^ but upto 15^th^ this month we could consider timely reporting. The server is not functioning, I donot know what to do.

I: So, I wonder since being locked, when will it be open? Could this month data not be reported?

R: I think it could be entered once it is opened. It will open. There might have been some problem with the server.

I: So, data is not locked?

R: Yes, I have also followed with higher authority. They said “data is not locked. There might be some changes in the server which had interrupted the functioning. We hope it will resolve soon.” Thus, from the official place such suggestion came to wait and try. I have been trying. They are informed about the issue here. So, it might be considered. I am sure.

I: Due to availability of features like validation and notifications during wrong or double entry, has it supported improvement of data quality?

R: Data quality is one thing but we have been receiving higher targets. Though we have been working continuously to meet target, we are unable due to it being higher. The target is set by higher authority as previous. If we could set our targets according to the population of our own RM, we could meet the targets and the conditions will have been improved. Some problems arise due to target being set by the center.

I: Do you have any experiences of observing large data in the DHIS-2 platform although you had entered only a small of them?

R: No sir. Only those we enter are stored there. No any such experiences.

I: There are examples like those data are in HMIS form but not included in DHIS-2 platform and we cannot enter. Have you faced any such issues?

R: Yes, sir! We have also faced those. The 9.2 form of HMIS and DHIS-2 are not synchronized in some. The higher officials said that it was due to typing errors and some software issues. They directed us to remember/note those and said to enter in those specific areas. (9:00 to 9:45)

I: Where are the utilization of data from your perspective?

R: Talking about utilization, they are used for sharing/progress reporting at the RM. We could easily share by opening the portal about the progress and coverage of our HF and RM. So, it has been easy.

I: have you practiced displaying charts, graphs, tables for the coverage, trend and progress inside your health facility?

R: The programs of health insurance is being started through this baisakh and jestha in here. So, it will be functioning through Bhadra month. Thus, after being functioning, they might direct us to fill those tables as well and send the required materials as well. Then we can do all sir.

I: My concern is their practice of displaying the progress report, trend of health issues in the HF?

R: Yes, sir! We have placed such data/messages in our information board of our office. We have also hanged the name list of HFOMC members, Health workers supervisory flowchart, FCHVs contact list, program wise listing of health workers. The information on delivery coverages and incentives provided.

I: How have the data been utilized for finding the disease burden in the community and the health promotion/awareness activities in the community level based on those data?

R: Yes, that are being done. The issues are raised in the meetings as well. We receive targets and work based on that. Though we do not have malaria in Mustang district, we receive targets for malaria. We also focus on those and work accordingly sir.

I: Have these data been utilized/ based on those data achieved, are there been the performance appraisal of your own or health facility through the palika?

R: For this time such data has not been used in performance appraisal or evaluation. Before performance appraisal was conducted bi-annually. But I must say, data are being utilized. In the meeting as well, they direct us towards achieving for the target and also appreciate the works done.

I: While formulation of plans and programs of health sector at the palika level, how are the decision made? How fruitfully the data are being utilized for decision making and planning?

R: Plan formulation and budget order are made based on the data nowadays. As, there is not push system now. The pull system is active and many activities are conducted based on data as well sir.

I: Can you provide some examples of such activity done you being involved in HF or done through palika level?

R: Could you clear more sir?

I: Could you report any examples you had or the palika had conducted based on the gap seen on the data or using the data?

R: I cannot say you right now.

I: What motivates you in operating the DHIS-2 software sir?

R: So, first thing is it is my duty to report the activities conducted and services provided. Thus, I must use this software. If I do not enter and report, how could I say I have worked and accomplished the tasks. This is the main motivation that we can show our progress and achievement through online.

I: How would you see the future of those online software in recording and reporting? Should they be continued, improved and used?

R: Yes, sir! They should work continuously. I would also recommend for improving the service targets by being based on the exact population of ward and palika but target should not be set from central level being based on estimation.

Channel/ system should be made such that we can continuously collect data and review those while planning for child health, safe motherhood based on the expected pregnancy, no. of child, no of mothers/women. We can find the exact data at the palika level, set the targets for services then only the achievement would be actual and reliable.

I: How interested do you find the people’s representatives regarding the use of software for recording and reporting of the services?

R: Yes, they are interested. We had received a program from province level to maintain the family profile through online platform. So, officials from province came and brief about the importance of family profile to the Palika chairperson, assistant chairperson and ward presidents. So, they get motivated and interested as they were more clear that we can easily observe the data on coverage and profile through online platform. We can get the data we need of the ward, palika or district which we had entered.

I: So, in the ward level as well there are sharing on data and related things?

R: I must say yes, we share. The main role with it is co-ordination. The co-ordination has improved nowadays. As the ward president is also the chairperson of our HFOMC. There are sharing and discussion on the health issues at ward level. We have meetings and coordination nowadays is also better than previous.

I: I found that you have not received training on DHIS-2. So, basic training is required at first. How do you feel the requirement of training for operating the DHIS-2 software or HMIS?

R: SO, for that there are much trainings. The HMIS, LMIS training package are also there. While in the training there, we had learnt an hour on DHIS-2 entry and data extraction only. The training should be organized based on the need and requirement of health worker; who is responsible for entry, extraction and analysis should be trained first on all the required functions that need to be known by the operator on DHIS-2.

Training should be focused on data interpretation, data quality assessment and assurance, what is KAS signal what is its function. These are the most important ones and required ones to operate the software.

I: Are there any training guidelines or training booklets in the health facility regarding DHIS-2?

R: Rather than HF these materials should be on Health office. The DHIS-2, HMIS, LMIS materials and packages are available on the health office in my view.

I: Are there any such materials available on the HF to study regarding operation of DHIS-2?

R: No sir. There are not any.

I: What will be the recommendations and suggestions on utilization of data from DHIS-2 in the Gandaki Province?

R: At first all health workers at all HFs should be aware on what is DHIS-2 and what are its functions. So, all health workers should be trained to operate DHIS-2. Some HFs might not have their ID till now. They do not even know where and how to enter the data. Thus, they should be trained first. After that the problems and errors arising while operating software should be addressed accordingly.

I: Could you add some more?

R: Um, yes sir. It has not been more since we started operating DHIS-2. Its been almost a year while before RM used to run the software. We entered in the record forms and submit to RM and the focal person used to enter into the software. But after training on HMIS I also started entry from here in the HF.

I: How could the data utilization be improved?

R: for that we should understand the nature of data; the (10-12) packages with in the software where, how to open, how to run them? After that we can use the data accordingly.

I: Any further additions and suggestions please?

R: Um yes, DHIS-2 is very good software to use. The training should be provided before installing in the HFs.

Previously, we had to work manually, record in the paper based forms, prepare so many charts manually for presenting in the review workshops as well. It was an arduous task. But now we can easily access up to which time period and present accordingly the progress. So, now it should be focused on improving the software more and address the errors/problems within it.

Thank you

Interview ended
