# Supplementary material for: Health professionals’ experience on District Health Information System (DHIS2) and its utilization at local levels in Gandaki province, Nepal: A qualitative study
Source: PLOS Glob Public Health. 2024 Mar 27;4(3):e0002890. doi: 10.1371/journal.pgph.0002890 (PMC10971587; doi:10.1371/journal.pgph.0002890)
Supplement: S6 Text — (DOCX) [file pgph.0002890.s007.docx]

**IDI_DHIS2FP_PHD_Gandaki**

Gandaki province DHIS2 Focal person

Statistical officer

Male

I: What is the situation of DHIS-2 in Gandaki province?

R: In the previous year, the situation of reporting was 100% while timeliness of reporting was nearly 85%.

I: What is the coverage and is it strengthened to all the health facilities?

R: We are unable to cover all the facilities. While all the palikas are using this DHIS. Also, in this year we gave 5 batches of training to the health workers regarding DHIS data entry. While looking at the reporting status nearly 500 institutions are doing data entry of 9.3 or 9.4 of DHIS. All the hospital (government and private) and other health institutions also use DHIS.

I: What are main problems faced by the health facilities and the palika level? What kind of problems do they share to you?

R: The main problem faced by all is the high traffic in the website or large numbers of users. Previously, it was only accessed by the district and palika level but now all the health facilities are using the DHIS. Due to which during the 5-10 of each months the server down issue arises very often. To solve this problem the I-HMIS section of the central government is working to increase the space of the DHIS and is in the phase of improvement.

I: Are there any data loss chance? Or sometimes due to the fault in DHIS the entered data is shown high or less than the original data?

R: I have also faced some of the problems sometimes. For example: A problem arise at at syangja. Some of the health workers despite of clicking the HMIS.gov.np/HMIS to enter their data, they mistakely enter their data at the DEMO and search at the live server. Which created problem in some of the places. However, under my experience I have not faced the problem of data loss till now. Nevertheless, sometimes due to the weak internet connection and ignoring the color indicators of internet i.e green, yellow and red, the data cannot be saved and might cause the data loss. Last year due to the internet issues and all 3 days of the data was lost but other than that there are no any experiences of data loss. Wrong URL is one of the reasons and we use any desk or phone calls to solve those problems.

I: Are you in frequent touch with the palikas/health facilities? Do they consult you frequently?

R: We are in lively touch with the 85 palikas they consult us frequently if any issues. Not only regarding the DHIS of HMIS, even the health workers are in frequent touch with us. Some of them prefer us more than the palika to consult at the time of problems. We have shared our contact information with them. And they consult us through the phone contact at the time of problem.

I: Is there mismatch in the entry forms?

R: In the previous year the HMIS section chief in support with the developmental partners revised the HMIS form which was not included in the program of AWPB, Red book. After it was finalized, we sent it for printing but later on at other workshops we realized there were problems in the form . If we could not tender the HMIS forms in Jestha-Ashad or Falgun-Chaitra we could not get the HMIS forms in Shrawan. The DHIS being electronic was revised according to the HMIS in Ashad. Still there are mismatch between the DHIS and HMIS hard copy forms. So, we informed all the health workers fill the form in accordance to the DHIS 2 and in the coming fiscal year we have revised the forms and probably the problems may not arise. In this year there is mismatch in main entry from, OPD and JC.

I: Some of the health facilities (Basic health care units and health post) are required to entry some services which is not available at the health facilities?

R: All the health facilities must enter their data within the 15 of each month. Talking about the new emerging health facilities for example, in some of the facilities there might not be any cases of malaria, leprosy. By chance if a case of the malaria came to the health facility, then in that situation it might be a problem. By common understanding and according to the type of health facility we have created and sent the data entry sets to the facilities. The health facilities must not take it as a burden. However, for example: sometimes mistakenly the data set regarding nutrition rehabilitation center can be allocated at the health facilities where the services are not available in that case, we immediately solve the problem.

I: Some data like family planning auto cumulates and creates a problem?

R: There is no such problem as auto cumulate but there is another problem like- while processing the data from the pivot table and exporting that to the excel sometimes there is a mismatch of data. I sent those mistakenly reported data to a gmail group which contains nearly 600 members (health workers of health facilities, palika and district). The DHIS system is designed in such a way that the previous month current user is the current year till the end of that month. For example: If there are 20 Depo users in Falgun than the current users in Falgun, Chaitra, Jestha and Ashad is also 20.

I: Can we separately get quarterly data?

R: This is a calendar issue. The meaning of quarterly in dictionary is ¼ i.e 20%. But in our government system the quarterly review means Shrawan, Bhadra, Ashoj and Kartik. It was supposed to be trimesterly but here while doing TB review and other we do it quarterly. This calendar issue is prevalent in our DHIS. While making pivot table four months can be selected manually and their values can be displayed. As we can see the values of a fiscal year properly, in quarterly we could not do that. If we could adopt the A.D. format there is no problem. But the countries where B.S is adopted there is a problem to assess the four months data properly. To solve this issue the data needed for the months can be selected manually and data could be gained.

I: What is the situation of infrastructure for DHIS 2 in Gandaki province?

R: There is a diverse geography in this province. There is no such problem in Pokhara, Kaski and even in lower Gorkha. But in upper Gorkha, and rural areas of Manang, Mustang there is not even the availability of mobile data. The geography has also created a problem. Analyzing the current information, we can say that 70-80% has DHIS infrastructures.

I: What is the situation of training and the motivation to use DHIS 2?

R: To provide the DHIS 2 training a budget is even allocated to the district. And also, some of the local levels has allocated their own budget to provide training. We also conducted 6 7 batches of training in this year. We are also planning to give training in the next year too. They also seem to implement what they have learnt in the training. Some of the health workers are not so IT friendly. While organizing the training we have tried to participate those who know to use laptop or computers.

Also, motivation is a qualitative thing. There is no such provision of motivation or punishment from the side of government but we should be hopeful and perform our duties. For example: a health worker took training of DHIS 2 but didn’t use it, in that case there is no provision of punishment. We have also exposed some of the health workers as a facilitator who have enough experiences and are motivated to use.

I: There is also complaint that health workers were only trained to enter the data and nothing other than that like functions like pivot table. Another complaint is only one health worker from a health facility was trained which created problem at the time of absent.

R: In the training organized by the Health Directorate, nearly 98-99% are trained to use the pivot table as well. Generally, we conduct training of 4 days and the training pattern has improved from the previous ones. Previously we have to depend upon the central server and internet. Now we have created our own local server due to which we should not depend upon external server and internet. In some cases, if the health workers are IT friendly than within 4 days of training the training could be revised for 2 times. Also, the training conducted by the district they often conduct training according to the need and demand. The integrated trainings containing HMIS, DHIS and e-LMIS is also conducted. The trainings are conducted from basic data entry to advance PIVOT table. These are conducted in the refresher training as well. We have also uploaded a tutorial video regarding PIVOT table in YouTube as well from where the health workers can update their skill.

I: The unavailability of training material or manuals for using DHIS was also reported by some of the health workers.

R: We are unable to provide the health workers with the hard copy of the training materials. There is no provision of providing these according to the budget and guideline. While providing IT training we have provided them with pdf or videos. We should not provide the manual on the pen drive as the manual is available in the DHIS 2 itself. The health workers must develop the practice to learn through the manual available in the DHIS.

I: Some health workers were not aware about the availability manual in the DHIS.

I: What is the situation of data utilization at Gandaki province?

R: When it comes to data, simply the data collection is not enough; the entire process of data cycle collection, processing, presentation, interpretation, and utilization needs to be completed for any meaningful outcome. If any step in this cycle is left incomplete, the system will not function properly. DHIS2 offers data for analysis, such as coverage and target versus achievement. To make use of this data, we hold an annual review meeting where we present and interpret the findings. These data are then utilized in the preparation of our annual work plan and budget.

Data utilization is existing as a national-level challenge, especially final use. The system emphasizes the importance of data use for decision making but its implementation is limited at various levels. Nevertheless, it is not feasible to rely entirely on data due to limited resources and unlimited demands that need to be met. Consequently. Adjustments must be made, but this does not imply a complete disregard for data usage of DHIS2.

Gandaki province has allocated budget for all the surveillance activities, in these activities we have used the DHIS2 data. Similarly, we used the DHIS2 data for full immunization declaration program. We have used the data in similar programs. However, I think we have not taken full advantage of the potential of using data to the extent that is necessary. We see some local levels some locals have started good initiatives by utilizing DHIS2 data.

Similarly, the utilization of data is also determined by the activeness of the staffs working at the planning level. The average number of individuals served by each immunization clinic is 45, and if certain health facilities exceed this number, they divide it into two separate clinics. If the data in DHIS2 indicates a range of 46-47, they split it into two clinics and utilize both. This is how they have utilized.

Similarly, they have utilized DHIS2 data to make various decisions regarding ORC clinics, safe motherhood, and reproductive health. However, the data has not been fully utilized to its maximum potential. There is a need to increase its utilization, and it requires collective effort from all of us.

I: Are there any activities from province like coordination with local levels and data literacy programs to enhance the use of data?

R: National statistical office is working on the data literacy or statistical literacy. As part of our efforts, we are also conducting a data literacy program for local elected representatives. During this program, it is important for us to use terminology and concepts related to the statistical literacy. We are doing advocacy on it. Recently, we organized an event where we invited president and deputy president of the Palika and discussed about the planning. However, it is not possible to fully literate them in a single day and developing data literacy takes time and requires a continuous effort.

Within the palika, we have a dedicated health section aimed at raising awareness among local elected representatives and other stakeholders about health data and related topics. The health workers working in this section have been facilitating this process. It is a gradual process, and we are making an effort to engage in discussions and consultations with stakeholders during our visits.

I: are DHIS2 data used for performance evaluation?

R: Yes, we have used data for performance evaluation. In a last fiscal year 2078/079, we employed DHIS2 data from all 85 local levels. After analyzing the data, we have ranked local levels from top to button. We recognized and awarded the top 10 performers among the local levels. We have also awarded to district health office and health sections of local levels.

During the annual review, we provide them with the indicators and assess their performance using these indicators. The entire process of evaluating performance and assigning rankings relies on data.

I: what is the situation of monitoring and supervision?

R: There are two things. Monitoring and evaluation are a continuous process and cannot be completed in a single instance. On the 16th-17th of each month, we check the data mismatch to determine, if necessary, elements are present or not. And we share it with 400-500 DHIS2 users and it helps to aware many of the users.

Likewise, our employees also conduct field visits to district health offices, local levels, and health facilities. During these visits, they examine the service registers of the health facilities and engage in discussions with their colleagues. Additionally, if necessary, we send letters to relevant parties. The DHIS2 platform incorporates various validations to ensure the quality of data, and we utilize it to demonstrate data quality during training sessions. Similarly, we have implemented validation rules within the DHIS2 system.

And the process of monitoring and evaluation is ongoing. Currently, we are transitioning towards utilizing electronic health records. Until now, we have been providing feedback by reviewing the DHIS2 system, specifically versions 9.3 and 9.4. However, it is not feasible for us to physically visit all health facilities to observe their health service registers. Nevertheless, the district health office and local levels are able to access these facilities and perform monitoring activities. In addition, we have conducted a trial of electronic health records in Galkot municipality, where all the records have been digitized. Earlier this morning, a case was registered at a health facility, and we contacted the facility to confirm the registration. Once confirmed, we inquired if the case was related to fever and if they had conducted a malaria test. We monitor cases in a similar manner. Once we implement electronic health records, we will be able to monitor data quality and provide feedback remotely. It is important to note that DHIS2 primarily focuses on reporting forms rather than recording forms, as it functions primarily as a reporting tool.

Pokhara metropolitan has also piloting electronic health records in different health facilities and the software they have developed has received approval from IHMIS section.

I: Many of the respondent have expressed their dissatisfaction with the absence on a section in DHIS2 to record and report data on locally implemented programs, in addition to the national health programs. Have any measures been taken to address these concerns?

R: After the arrival of elected representatives, various schemes and services have been introduced to demonstrate their involvement in the community and to ensure that the government is actively working to address their needs. However, there is currently no provision in DHIS2 to record and report all the activities carried out by local levels.

When discussing the activities carried out by them, the nature of the program is different in different palikas. There are differences between municipalities within the Gandaki province. For instance, the approach to elderly programs differs from one palika to another. Our current system manages to capture some aspects, but it fails to capture all aspects of the program as per their desire.

We do not have a system for recording and reporting similar to DHIS2, but we have offered a platform for them to share their activities and their contributions to the health system. Recently, we organized an event where we invited around 400-500 participants, including Mayer, vice-Mayer, president, vice president, and health coordinators to share their innovative works in the health sector. However, like you said, we can’t change or revise the HMIS system for Gandaki province because it has been implemented across Nepal.

As previously mentioned, if there are differences among the various local levels within the Gandaki province, there are also variations among different provinces. Likewise, the target groups differ as well. These activities need to be incorporated into the system since they are integral to it. We have been trying to include these things by developing any mechanism. However, it should have not been developed.

I: in some countries there is a practice of publishing bulletins including success stories and best practices on DHIS2 performed by health facilities so other health facilities can learn from them. Are there such practices or if you have any plans?

R: We don't only create bulletins specifically for DHIS2, but we produce bulletins that encompass DHIS2 along with other health service programs and related activities. We release weekly and monthly bulletins through the provincial health directorate, where we highlight DHIS2 activities as well as other initiatives carried out in this field. Similarly, district health offices and other provincial offices also publish monthly bulletins. If you refer to the monthly bulletin of Baglung district, you will find information about DHIS2 activities and other health-related activities.

While the published reports don't currently include case stories or success stories as you mentioned, they do contain information pertaining to DHIS2. However, we agree that incorporating such stories would be beneficial and should be considered in the future.

I: What additional strategies is the provincial health directorate implementing for DHIS2, and what efforts are being made to enhance its usage?

R: The Gandaki province intends to enhance the electronic health record system in the near future, which has already been implemented in certain healthcare facilities. In terms of data accuracy, the issue arises when transferring data from one service register to another or during the tallying process, resulting in misplaced data.

After making the service register electronically using software, the system automatically controls the data quality. So, we have decided to transition to electronic health records, which is now the top priority program of the Gandaki province Ministry of Social Development and Health.

The Health and Population ministry has provided a list of game changing programs, and digitalization is included as one of them. Therefore, it is crucial that we give it high priority and proceed with its implementation.

Regarding the utilization of data, as I mentioned earlier some evidences, we can increase the data utilization by applying these evidences. Simply gathering the data without completing the data cycle will not yield any results.

Until the data have not been used, till not applied in utilization, they will store only and not used. We must enhance the utilization of data, employing it for the preparation of AWPB (Annual Work Plan and Budget) and for making daily decisions. We need to link it with human resources, finance, and logistics. The WHO has identified six essential components of a health system, with information being one of them. Therefore, it is important for us to establish links with the other five components as well. And we have a plan for this and we have also added it into our policies and programs.

I: what do you think it is better for the eLMIS, DHIS2, and eTB to be integrated or kept separate?

R: We have a HMIS roadmap, which clearly mentioned the integration of different information systems. However, there is a need to focus on establishing a robust server and ensuring skilled human resources. The integration process will be gradual, such as when implementing the Electronic Health Record (EHR) software, where the electronic Tuberculosis (eTB) system will be incorporated into it. This integration will allow for the inclusion of the TB treatment register within the EHR, facilitating easy data extraction. Similarly, the logistics system will also be integrated with the EHR. It is imperative to adhere to the roadmap's guidelines, as all the systems will ultimately be merged into a unified platform.

The required things and the processes we must take require a significant amount of time. While it may not be feasible to achieve today, it will be completed within a few years and it imperative that we undertake it

I: Thank you

R: Thank you

End
