# Supplementary material for: Health professionals’ experience on District Health Information System (DHIS2) and its utilization at local levels in Gandaki province, Nepal: A qualitative study
Source: PLOS Glob Public Health. 2024 Mar 27;4(3):e0002890. doi: 10.1371/journal.pgph.0002890 (PMC10971587; doi:10.1371/journal.pgph.0002890)
Supplement: Attachment — Submitted filename: Reviewer response PGPH PGPH-D-23-01750.docx [file pgph.0002890.s009.docx]

Reviewer response PGPH-D-23-01750

Overall comments:

This is an important study and has interesting qualitative findings.

I would recommend that the authors consolidate some of subthemes further as some seem to be related to each other. I think another strong theme is DHIS2 QATA quality or accuracy.

In the discussion, further synthesis is needed to indicate how the findings add to the base of evidence - contributions to or advances to the literature.

What is the overall, crosscutting theme that encompasses all the themes and subthemes?

What is the theoretical framework that guided data collection and analysis?

Was this a grounded theory approach – you mentioned reaching saturation?

Presentation of the themes and subthemes could be improved – perhaps 4 tables for each theme and subtheme. As it is right now, it is hard to keep track of what theme is being described.

Specific comments:

In the abstract please indicate the period during which the study was done

Rephrase “incompetent human resources” in abstract and Introduction (para 3)

Of note are other concerns about the quality of data uploaded to the DHIS2 e.g., in Kenya – a study I conducted found low data quality and completeness (https://bmchealthservres.biomedcentral.com/articles/10.1186/s12913-023-10133-2). So DHIS2 implementation is not a direct guarantee of accurate and reliable implementation in some settings.

You accurately highlight the issue of dual data entry (manually and electronically) is this a challenge for the health professionals you encountered in your study?

Which health professionals or cadres use the DHIS2? Is it primarily doctors or nurse.

Which approach was used to develop the IDI guide? Please be clear – whether it was grounded theory or not?

Please provide more detail about the development of the indepth interview guide – was this guided by a conceptual framework or theory focused on HMIS? You need to use a holistic framework to ensure that your analysis is robust and strong. Please indicate if a conceptual framework guided the research question, data collection and analysis? If not, please explain why not?

How were the data pretested before collection?

Was there a validation exercise or some form of member checking (i.e., with stakeholders) that was done prior to use of the tool

Please define the abbreviations Sr. AHW, Sr. ANM, AHW, and ANM. Were these doctors, nurses?

Out of how many hospitals or facilities were the participants sampled?

Were the public health officers overseeing more than one hospital. Are they direct users of DHIS2? Please explain their level of interaction with DHIS2 whether they see patients, input data or use data for planning? At which point do they interact with the system?

Please be specific – you mention in methods that you “reviewed several times”? Specifically, how many times were transcripts reviewed?

How were the data secured? Were they anonymized?

Why were findings not shared with participants after the analysis, e.g., for member checking?

Table 1 – please provide definitions for abbreviations as footnotes.

The presentation of the themes and quotes can be improved for example by using tables so that it’s reader friendly.

Are there specific efforts to assess the quality of data in the DHIS2 prior to its utilization for planning and forecasting.

Consider issues of information security as a concern or challenge were the health professionals have to take registers and computers home to upload data when the internet service is better. Is this something you explored? If not, perhaps mention as a limitation.

Have you considered data quality of DHIS2 as a potential subtheme given some of the comments highlighted? Perhaps rewording the subtheme on “Error monitoring and feedback” and some overlap with this for “System issues” subtheme with data mismatches. I see this crosscutting with other themes like “data entry and analysis”, “support in data analysis.

The issue of data loss is important and could be more strongly highlighted in your discussion. Esp with considerations or recommendations for improving DHIS2 to be more stable and resilient to internet instability etc.

Consider adding theme of “Solutions” in addition to “Opportunities” i.e., “Opportunities and potential solutions”

Instead of the term of “competency”, please use more standard and acceptable terms like “self-efficacy” or “user experience”—or lack thereof

You have the data analysis subtheme under more than one theme. Please synthesize your subthemes and themes further. It is possible to distill what you have into at least 4 themes each with no more than 4 subthemes. There is a lot of repetition in the thematic areas in the results section.

Please provide one quote per theme to reduce the word count. Like I mentioned above, you will need to consolidate, reduce the subthemes and put the themes & subthemes in tables.

I recommend English copy-editing services to improve the grammar and flow of the paper.

Discussion section should be trimmed down – please remove subheadings for better flow. What is your overall theme or primary take away from your findings? Please do not merely repeat the findings from the results section in the discussion but rather synthesize your findings and discuss where your findings sit in the literature (what is new, different from, or similar to the current literature). The discussion section should be a streamlined critical analysis of your take on the findings and the contributions to the wider field.

Limitations – please include other limitations of the qualitative design, e.g., lack of generalizability etc.

Conclusion – could be stronger and more summative of your findings. It is currently a repetition of the results. Please be more critical and thoughtful of your overall message and what the take away message will be from this research.

Please remember to update the COREQ checklist after making revisions.
