# Supplementary material for: Health professionals’ experience on District Health Information System (DHIS2) and its utilization at local levels in Gandaki province, Nepal: A qualitative study
Source: PLOS Glob Public Health. 2024 Mar 27;4(3):e0002890. doi: 10.1371/journal.pgph.0002890 (PMC10971587; doi:10.1371/journal.pgph.0002890)
Supplement: Attachment — Submitted filename: Response to Reviewers.docx [file pgph.0002890.s010.docx]

**Response to Reviewers**

Dear Editor and Reviewers,

We would like to express our sincere gratitude to you for devoting your quality time to reviewing our manuscript and offering us detailed input which has been extremely helpful in shaping our manuscript. Below are our responses and actions corresponding to your comments.

**Comment 1: What is the overall crosscutting theme that encompasses all the themes and subthemes?**

Response: Thank you for your comment. The overall crosscutting theme that encompasses all the themes and subthemes is the **“Utilization of DHIS2 by health professionals at the local level”.**

**Comment 2: Was this a grounded theory approach – you mentioned reaching saturation?**

Response: The study did not adhere to the grounded theory approach. Nevertheless, based on the literature, saturation is a benchmark for determining sample sizes and ensuring rigour. Hence, the study incorporated the concept of saturation. <https://www.tandfonline.com/doi/pdf/10.1080/23311886.2020.1838706>

<https://www.ncbi.nlm.nih.gov/pmc/articles/PMC5993836/#:~:text=In%20broad%20terms%2C%20saturation%20is,data%20collection%20and%2For%20analysis.&text=Its%20origins%20lie%20in%20grounded,of%20approaches%20to%20qualitative%20research>.

**Comment 3: Presentation of the themes and subthemes could be improved – perhaps 4 tables for each theme and subtheme. As it is right now, it is hard to keep track of what theme is being described.**

Response: Thank you so much for your comment. I have restructured the table in the revised manuscript.

**Comment 4: In the abstract please indicate the period during which the study was done**

**Rephrase “incompetent human resources” in abstract and Introduction (para 3)**

Response: The abstract adds the study duration and rephrases the word.

**Comment 5: You accurately highlight the issue of dual data entry (manually and electronically) is this a challenge for the health professionals you encountered in your study?**

Response: According to the participants, they must prepare a monthly report for entry in DHIS2. Therefore, they advocate for the implementation of an electronic data entry system.

**Comment 6: Which health professionals or cadres use the DHIS2? Is it primarily doctors or nurse.**

Response: In Nepal's health system, there is a designated person responsible for utilizing DHIS2 at all health facilities. For instance, at the hospital level, there exists a role known as the medical recorder or statistician, and individuals in these positions are the primary users of DHIS2. In cases where such a position is not present, paramedics or nursing staff take on the responsibility. Likewise, this research was carried out at the local government and health post levels. At the local level health section, a trained and experienced focal person, such as a health assistant, auxiliary nurse midwife, or auxiliary health worker, is in charge of DHIS2. Similarly, at the health post level, DHIS2 is managed by trained and experienced staff members like health assistants, auxiliary nurse midwives, and auxiliary health workers.

**Comment 7: What is the theoretical framework that guided data collection and analysis?**

**Comment: Which approach was used to develop the IDI guide? Please be clear – whether it was grounded theory or not?**

**Comment: Please provide more detail about the development of the in-depth interview guide – was this guided by a conceptual framework or theory focused on HMIS? You need to use a holistic framework to ensure that your analysis is robust and strong. Please indicate if a conceptual framework guided the research question, data collection and analysis? If not, please explain why not?**

Response:

Thank you. We opted not to rely on frameworks or theories derived from the health management information system. The nature, objectives, and context of our study influenced this decision. Similarly, since our objective was to examine diverse perspectives among health professionals regarding the utilization of DHIS2, we abstained from integrating any frameworks or theories.

Nevertheless, a comprehensive literature review and discussions with experts in DHIS2 and HMIS were conducted while developing in-depth interview guidelines. Similarly, the research process was meticulously crafted to ensure rigour, coherence, and alignment with the study objectives.

We are confident in the robustness of our data collection and analysis strategies, maintaining methodological coherence throughout the study. This flexibility enabled us to uncover valuable insights that might have been overlooked within a more rigid framework.

**Comment 8: How were the data pretested before collection?**

Response: We pretested the data both at the local level and health facility level before the collection. Additionally, discussions were held with DHIS2 focal persons at the provincial and local levels, who reviewed the guidelines. Mock sessions were conducted with authors and local health professionals to ensure the clarity and flow of the questions.

**Comment 9: Was there a validation exercise or some form of member checking (i.e., with stakeholders) that was done prior to use of the tool**

Response: Yes, the tool underwent validation through collaboration with provincial DHIS2 focal persons who provided feedback. Additionally, the tool underwent review by a local-level focal person before its utilization.

**Comment 10: Please define the abbreviations Sr. AHW, Sr. ANM, AHW, and ANM. Were these doctors, nurses?**

Response: These are the community-level health workers. The abbreviations are added in the manuscript. Here are the abbreviations

Sr. AHW – Senior Auxiliary Health Worker

Sr. ANM – Senior Auxiliary Nurse Midwife

AHW - Auxiliary Health Worker

ANM – Senior Auxiliary Nurse Midwife

**Comment 11: Out of how many hospitals or facilities were the participants sampled?**

Response: In the Gandaki province of Nepal, 11 districts comprise 85 local levels. Five districts, representing mountainous, plain Terai, and hilly areas, were specifically chosen due to their distinct geographical characteristics. Within these selected districts, one metropolitan, sub-metropolitan, or urban municipality and one rural municipality were chosen, resulting in two local levels selected from each district. The study included a health section and a health facility at the local level. Consequently, two participants were chosen from one local level, totalling four participants from each district and 19 from the local levels overall. However, one participant from the mountain region did not meet the inclusion criteria, leaving the final participant count at 19. Additionally, the DHIS2 program operates from the provincial health directorate office, necessitating the inclusion of one focal person from the provincial health directorate office in the study.

It's important to note that the study focused solely on the local level and health post level, excluding hospitals. This limitation is explicitly acknowledged in the study.

**Comment 12: Were the public health officers overseeing more than one hospital. Are they direct users of DHIS2? Please explain their level of interaction with DHIS2 whether they see patients, input data or use data for planning? At which point do they interact with the system?**

Response: Public Health Officers operate within public health facilities rather than hospitals, as outlined by the current health system structure. Their positions are authorized at urban municipalities and higher institutions. In predominantly supervisory roles, they monitor and review DHIS2 data reported from health facilities, offering feedback and guidance to health workers on DHIS2 utilization. Although not directly involved in data entry, they may enter data at the municipality level under specific circumstances, such as when health facility staff need more skills or are absent during monthly reporting periods. Moreover, public health officers contribute to planning and decision-making at various levels, including the health section of the local level, district health office, and provincial health directorate office.

**Comment 13: Please be specific – you mention in methods that you “reviewed several times”? Specifically, how many times were transcripts reviewed?**

Response: Addressed in the revised manuscript.

**Comment 14: How were the data secured? Were they anonymized?**

Response: Addressed in the revised manuscript.

**Comment 15: Why were findings not shared with participants after the analysis, e.g., for member checking?**

Response: We shared the findings with the participants, seeking their input for review. Unfortunately, despite my follow-up attempts, we didn't receive any response from them. As a result, member checking was not conducted in this study. Nevertheless, the interviews were audio recorded, notes were taken, and the data were transcribed and translated.

**Comment 16: I would recommend that the authors consolidate some of subthemes further as some seem to be related to each other. I think another strong theme is DHIS2 QATA quality or accuracy.**

Response: We have tried to consolidate themes and subthemes and repetitions of information across the themes and subthemes.

**Comment 17: Table 1 – please provide definitions for abbreviations as footnotes.**

Response: Added in the manuscript.

**Comment 18: The presentation of the themes and quotes can be improved for example by using tables so that it’s reader-friendly.**

Response: We tried to put our quotes in a table, but the quotes were too big, making the table too large. So, we decided not to include the table. If you think it's important to have a table with quotes, please tell us, and we'll make the changes needed.

**Comment 19: Are there specific efforts to assess the quality of data in the DHIS2 prior to its utilization for planning and forecasting.**

Response: The majority of respondents emphasized that monthly, semiannual, and annual review meetings take place at the local, district, and provincial levels. These meetings serve as the foundation for evaluating the quality of DHIS2 data before engaging in planning and forecasting activities.

**Comment 20: Have you considered data quality of DHIS2 as a potential subtheme given some of the comments highlighted? Perhaps rewording the subtheme on “Error monitoring and feedback” and some overlap with this for “System issues” subtheme with data mismatches. I see this crosscutting with other themes like “data entry and analysis”, “support in data analysis.**

Response: Addressed in the findings.

**Comment 21: Consider adding theme of “Solutions” in addition to “Opportunities” i.e., “Opportunities and potential solutions”**

Response: Thank you for your suggestions. Added in the finding.

**Comment 22: Instead of the term of “competency”, please use more standard and acceptable terms like “self-efficacy” or “user experience”—or lack thereof**

Response: Addressed in the manuscript.

**Comment 23: You have the data analysis subtheme under more than one theme. Please synthesize your subthemes and themes further. It is possible to distill what you have into at least 4 themes each with no more than 4 subthemes. There is a lot of repetition in the thematic areas in the results section.**

Response: We have tried to remove the repetitions in the thematic areas in the findings. The subthemes have been reorganized by combining duplicated information, and the overall number of themes has been reduced to six.

**Comment 24: Please provide one quote per theme to reduce the word count. Like I mentioned above, you will need to consolidate, reduce the subthemes and put the themes & subthemes in tables.**

Response: We tried to minimize the word count by consolidating duplicated information across themes and condensing the quotes to one per theme.

**Comment 25: Discussion section should be trimmed down – please remove subheadings for better flow. What is your overall theme or primary take away from your findings? Please do not merely repeat the findings from the results section in the discussion but rather synthesize your findings and discuss where your findings sit in the literature (what is new, different from, or similar to the current literature). The discussion section should be a streamlined critical analysis of your take on the findings and the contributions to the wider field.**

Response: Thank you. As per the suggestions, we have made the necessary corrections to the discussion part of the revised manuscripts.

**Comment 26: The issue of data loss is important and could be more strongly highlighted in your discussion. Esp with considerations or recommendations for improving DHIS2 to be more stable and resilient to internet instability etc.**

Response: Added in the discussion part of the revised manuscript.

**Comment 27: please include other limitations of the qualitative design, e.g., lack of generalizability etc.**

Response: Added the limitation of the study in the revised manuscript.

**Comment 28: Consider issues of information security as a concern or challenge were the health professionals have to take registers and computers home to upload data when the internet service is better. Is this something you explored? If not, perhaps mention as a limitation.**

Response: We didn’t explore any details on this issue. We appreciate your feedback, and we have mentioned it in the limitation in the revised manuscript

**Comment 29: Conclusion could be stronger and more summative of your findings. It is currently a repetition of the results. Please be more critical and thoughtful of your overall message and what the take away message will be from this research.**

Response: We tried to revise the conclusion in the revised manuscript.

**Comment 30: Please remember to update the COREQ checklist after making revisions.**

Response: Updated.
